# Chlordecone: development of a physiologically based pharmacokinetic tool to support human health risks assessments

**DOI:** 10.1007/s00204-022-03231-3

**Published:** 2022-02-05

**Authors:** Claude Emond, Luc Multigner

**Affiliations:** 1 PKSH Inc., Mascouche, QC Canada; 2 School of Public Health, Département de Santé Environnementale et Santé au Travail (DSEST), University of Montreal, Montreal, QC Canada; 3 Univ Rennes, Inserm, EHESP, Irset (Institut de recherche en Santé, environnement et travail)-UMR_S 1085, Rennes, France

**Keywords:** Chlordecone, French West Indies, PBPK, Pesticides, Pharmacokinetic

## Abstract

Chlordecone (CD; Kepone™) is a carcinogenic organochlorine insecticide with neurological, reproductive, and developmental toxicity that was widely used in the French West Indies (FWI) from 1973 to 1993 to fight banana weevils. Although CD has not been used there for more than 25 years, it still persists in the environment and has polluted the waterways and soil of current and older banana fields. Today, human exposure to CD in the FWI mainly arises from consuming contaminated foodstuffs. The aims of this study were to develop a physiologically based pharmacokinetic (PBPK) model in the rat and extrapolate it to humans based on available pharmacokinetic data in the literature. A comparison of simulations using the rat model with published experimental datasets showed reasonable predictability for single and repetitive doses, and, thus, it was extrapolated to humans. The human PBPK model, which has seven compartments, is able to simulate the blood concentrations of CD in human populations and estimate the corresponding external dose using the reverse dosimetry approach. The human PBPK model will make it possible to improve quantitative health risk assessments for CD contamination and reassess the current chronic toxicological reference values to protect the FWI population.

**Supplementary Information:**

The online version contains supplementary material available at 10.1007/s00204-022-03231-3.

## Introduction

Chlordecone (CD) (CAS: 143.50.0), also known as Kepone™ or Curlone^®^, is an organochlorine insecticide that was used in the French West Indies (FWI) from 1973 to 1993 to fight banana weevils (Multigner et al. 2016). Although CD has not been used in the FWI for more than 25 years, it is still present in the soil due to its very slow degradation in the environment (Cabidoche 2009). Such pollution affects approximately one-third of the agricultural land in the FWI (Anses 2017), thus contaminating drinking water resources and vegetal and animal foodstuffs (Bocquene and Franco 2005; Dubuisson et al. 2007) and, consequently, most of the human population (Dereumeaux et al. 2019; Guldner et al. 2010; Kadhel 2008).

Many in vivo and/or in vitro experimental studies have found that CD is a neurotoxic, reproductive, and developmental toxicant and carcinogen in rodents and an endocrine disruptor (ATSDR 2020). The toxicity of CD in humans was first observed in 1975 after a poisoning episode involving CD manufacturing plant workers in Hopewell, VA, USA (Cannon et al. 1978). Workers were exposed to high CD concentration through oral, respiratory, and cutaneous routes. Such exposure resulted in several health disorders involving the central nervous system (tremors of the limbs), liver (hepatomegaly), and testes (reduced sperm production) (Cannon et al. 1978; Taylor et al. 1978). These clinical observations were regrouped under the term “Kepone syndrome” and mostly observed at plasma concentrations > 1 mg/L.

In the FWI, the general population is still continuously exposed through food contamination at lower CD concentrations than those to which the Hopewell workers were exposed. Epidemiological studies conducted in the FWI since 1999 have shown blood concentrations < 0.1 mg/L (Multigner et al. 2016). These studies showed that such levels of CD exposure are associated with long-term health disorders, including prostate cancer, prematurity, cognitive or motor development and epigenetic reprogramming in utero (Boucher et al. 2013; Kadhel et al. 2014; Legoff et al. 2021a; Multigner et al. 2010).

Various approaches have been used to assess exposure of the FWI populations to CD. One was to collect blood to determine the internal concentrations of CD (Kadhel et al. 2014; Multigner et al. 2010). Because of the long half-life (*T*_1/2_) in blood (between 63 and 165 days in humans) (Adir et al. 1978; Cohn et al. 1978), this approach covers all exposure routes and represents a good surrogate of the body burden at steady state (SS). Another approach was based on the estimation of dietary intake, combining food contamination with food consumption habits (Guldner et al. 2010; Seurin et al. 2012). However, both approaches have their limitations, which represent a challenge for human health risk assessments.

The chronic threshold limit value (TLV) currently used for CD risk assessment (0.50 µg/kg bw/day) is based on the most sensitive measurements of adverse effects observed in rats (Larson et al. 1979). Recently, The French Agency for Food, Environmental, and Occupational Health Safety (ANSES) revised the chronic external CD TLV (TLV_external_) using new available data (Gely-Pernot et al. 2018; Kadhel et al. 2014; Legoff et al. 2019). As a result, the chronic TLV_external_ was reduced to 0.17 µg CD/kg bw/day based on Human Equivalent Dose (HED) and uncertainty factors (Anses 2021). In addition, two chronic internal TLVs (TLV_internal_) were determined, one (0.47 µg/L plasma) based on the animal study of Larson et al. (1979) and the other (0.40 µL/L plasma) using the epidemiological study data during pregnancy of Kadhel et al. (2014) and Anses (2021). To obtain the chronic TLV_external_, the ANSES added an uncertainty factor for the TLV_internal_ from animal studies because of the absence of a human physiologically based pharmacokinetic (PBPK) model. Nonetheless, the two TLV_internal_ values are relatively close but do not consider the same approach or the same endpoint. A better alternative would be to develop a PBPK model for humans to obtain a better estimate of the external dose from internal doses by reverse dosimetry or appraise a predictive internal dose based on the ingestion of CD in food. The objective of this work was to develop a mathematical PBPK model in rats and extrapolate it to humans.

In the present study, we constructed PBPK models starting with a rat model and then extrapolated it to humans. Indeed, PBPK models are frequently developed from animal data because experimentation provides an opportunity to control and measure various pharmacokinetic parameters. Such models are then extrapolated to predict human tissue concentrations. Classical examples of successful scale-up from animals to humans have been reported in the literature, such as for styrene, dioxin, and methylene chloride (Andersen and Clewell 1987; Emond et al. 2016; Ramsey and Andersen 1984). For this study, all available publications concerning the pharmacokinetics of CD were reviewed to obtain the best hypothesis or assumptions possible.

## Materials and methods

### Pharmacokinetics of chlordecone in rats and humans

All mammals show similar CD pharmacokinetics, with some disparity (Guzelian 1982b). CD can be absorbed by inhalation, orally, and through the skin (ATSDR 2020). Occupational exposure involved the respiratory, oral, and dermal routes, whereas oral exposure via food consumption is the main route for the general population in FWI (ATSDR 2020). Most in vivo studies have explored the pharmacokinetics and toxicity of CD following oral exposure.

CD is readily absorbed (more than 90%) from the gastrointestinal tract (GIT) following oral exposure for all mammals studied. Due to its lipophilicity (Log Kow ~ 5.41), CD is then distributed between the portal vein and lymphatic circulation (De Winne 1979). The portal vein drains CD to the liver, where it undergoes the first passage. From the lymph, CD partially enters venous circulation. In the blood, CD is always found as the parent compound, which is largely carried by albumin and high-density lipoproteins (HDL) (Soine et al. 1982). HDL in the blood and lymph is associated with reverse cholesterol transport pathways (Reichl 1994; Skalsky et al. 1979a). In the liver, CD is partially reduced into the CD alcohol (CD-OH) by the chlordecone reductase (also called AKR1C4) present in humans, pigs, gerbils, and rabbits, but not significantly in rats, mice, or hamsters (Boylan et al. 1977, 1978; Guzelian 1982b; Molowa et al. 1986; Soine et al. 1983).

In rats, CD induces the microsomal enzymes of P-450, such as 7-ethoxyresorufin-*O*-deethylase and ethoxycoumarin-*O*-dealkylase, 3 days after a single oral dose of 15 mg CD/kg bw (Carpenter and Curtis 1991; Gilroy et al. 1994). No induction has been observed below 1 ppm of dietary exposure (Fabacher and Hodgson 1976). The ratio of CD to CD-OH in the liver varies from 1:1.3 to 1:3.9 in humans, whereas it is > 100:1 in rats (Houston et al. 1981). In humans, CD, as CD-OH and its glucuronide conjugate (CD-O-G) formed in the liver, is then excreted into the bile (Soine et al. 1983).

CD is also bound to hepatic proteins, called chlordecone binding proteins (CDBP) (Fariss et al. 1980; Soine et al. 1984). CDBPs concentrate the CD in the liver, resulting in an unusual apparent liver/fat ratio of 5 to 10 versus 0.3 based on theoretical calculations (GastroPlus 2018). This unusual ratio suggests that CD binds to cytosolic CDBP, which influences the elimination of CD through the bile, promoting the sequestration of CD in the liver (Soine et al. 1984).

The major route of elimination of CD for rats and humans is through the faeces (Guzelian 1982b). For rats, approximately 60% of CD is eliminated in faeces, versus 1.5% in the urine, 84 days after a single oral dose of 40 mg/kg bw (Egle et al. 1978). However, in humans, an estimated 10% of the CD in bile is expelled through faecal elimination (Cohn et al. 1976). Indeed, approximately 1% of the body burden is eliminated daily, but only 5% of the biliary elimination is accounted for in the faeces (Guzelian 1982a). This suggests that 95% of the CD is reabsorbed from the GIT (enterohepatic circulation) (Bungay et al. 1981). For humans, the CD-O-G excreted in bile is de-conjugated in the GIT and the resulting CD-OH mostly reduced to CD and then reabsorbed by the intestinal wall (Cohn et al. 1978; Scheline 1973). The literature also reports excretion from the blood to the GIT, increasing the elimination of CD in faeces for rats and humans (Boylan et al. 1979; Bungay et al. 1980). However, this enteric excretion fraction is not reabsorbed, suggesting that it takes place before the region of reabsorption. In rats, the blood *T*_1/2_ after a single oral dose of 40 mg CD/kg bw is 8.5 days (during the first 4 weeks), 24 days (from week 4 through week 8), 45 days (for weeks 14 and beyond) (Egle et al. 1978) and 18 days from another publication (Matthews 1979). In humans, the blood *T*_1/2_ reported in the literature is 165 days (Cohn et al. 1978), 96 days, with a range between 63 and 148 days (Adir et al. 1978), and 150 days (Guzelian et al. 1981).

### Previous PBPK models developed for rodents

Five rat PBPK models relating exposure to CD have been published (for more complete descriptions, see Supplementary Materials). These PBPK models do not allow simulations in terms of lifetime exposure or the enterohepatic cycle, which are essential pharmacokinetic components.

### Structural rationale, physiological parameters, and limitations of the PBPK model

**Fig. 1:**
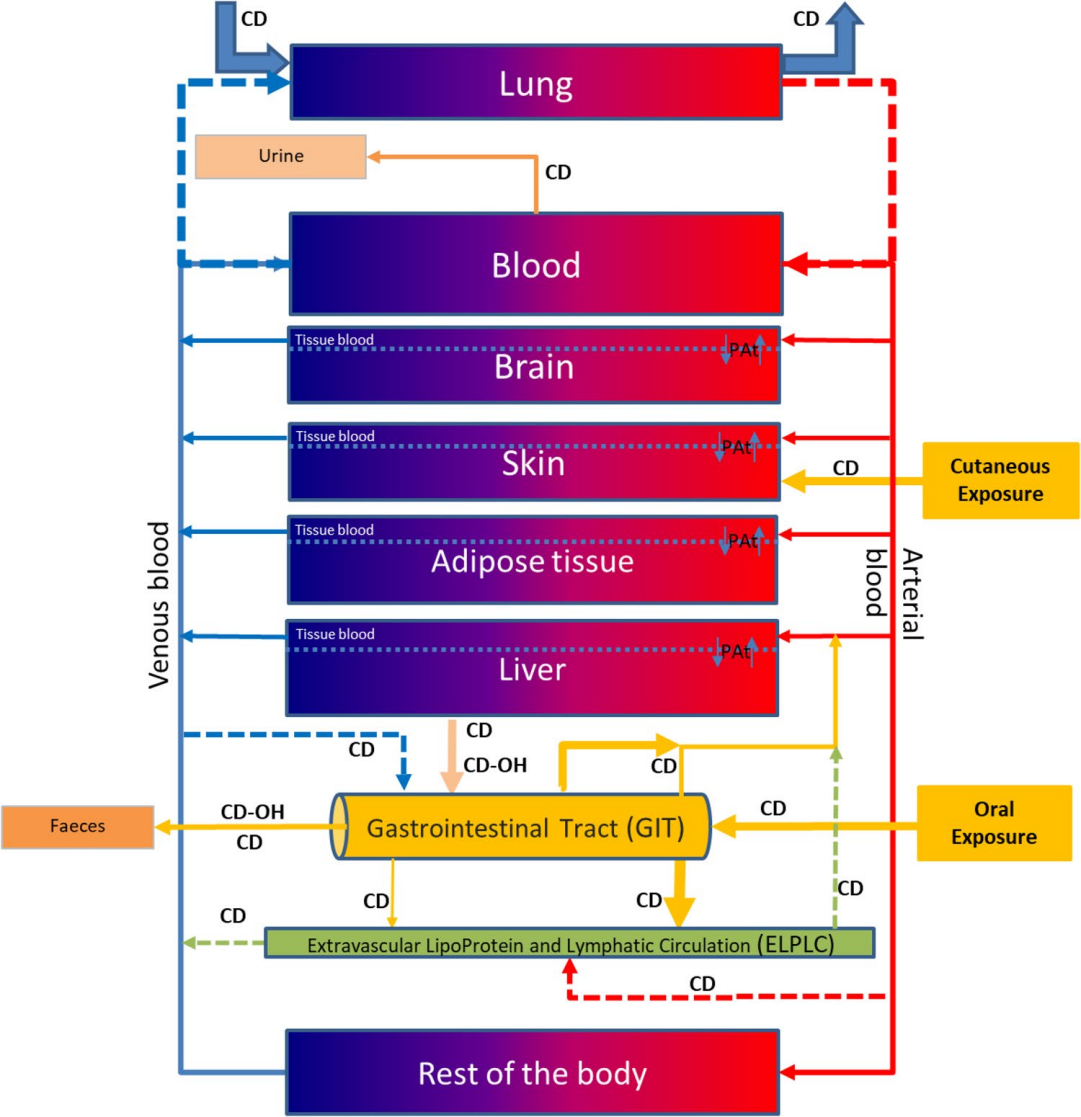
Conceptual representation of the PBPK model to study CD exposure in rats and humans. For rats, only CD is described, as there is almost none of the metabolite CD-OH. For humans, CD-OH is present as the major metabolite. The structure is the same for both species

The PBPK model (rat and human) we developed contains seven compartments: lungs, blood, brain, skin, adipose tissue, liver, and the rest of the body (RE), which corresponds to other tissues or organs not specifically described as compartments (Fig. 1). Each described compartment has a rationale:

- The Lung, Skin, and GIT compartments represent the routes of exposure. The literature has reported CD exposure via dermal and inhalation routes. Thus, these routes are described in the current PBPK model, although validation will require supplementary data. Oral ingestion, mostly from the diet, represents the major route of exposure. The GIT is described as a pseudo-compartment.
- The Blood compartment for the systemic circulation and protein binding (described as the percentage not affected by saturation). Only free chlordecone (CD_free_) can leave the blood to enter other compartments.
- The Brain compartment because it is a target of organ toxicity.
- The Adipose tissue compartment because of the lipophilicity of CD.
- The Liver compartment is the major site of metabolism and storage, contributing to the biliary elimination of CD, CD-OH, and CD-O-G in humans and the enterohepatic cycle in humans and rats.
- The extravascular lipoprotein and lymphatic circulation sub-compartment (ELPLC) includes communication with the GIT, portal vein, and veins and arterial blood, which is important as CD follows the reverse cholesterol pathway via HDL according to the literature.
- The rest of the body compartment (RE) is described to maintain the mass balance.

All compartments, except the RE and lung have been reported to show permeability-limited distribution, suggesting that CD slowly diffuses between the sub-compartments of the tissue blood and cellular matrix, requiring time for diffusion. The ELPLC is part of the RE (Fig. 1). For a better visual description, the ELPLC is shown separately extracted from the RE compartment in Fig. 1. This PBPK model was rigorously developed following the anatomical, physiological, and pharmacokinetic data from the literature. Both the rat and human models were used to determine lifetime exposure. The elimination half-life assumed for the models is 21 days for the rat model (based on the range of 18 and 24 days reported in the literature) and 131 days for the human model (corresponding to the mean value of 96 days and 165 days provided in the literature).

### Parametrization of the model (rats and humans)

Parameters of the PBPK model (e.g., volumes of the compartments, body weight) were determined based on age using polynomial equations (Luecke et al. 2007; USEPA 2010). All equations and descriptions of the parameters are presented in the Supplementary Materials. To simulate anatomical and physiological parameters in rats and humans at any age, whole-life polynomial equations were included in the model (regardless of the blood *T*_1/2_ of a chemical). The cardiac output and alveolar ventilation rate were calculated based on the body weight of the species (Krishnan and Andersen 2008) (Supplementary Table S1). The partition coefficient was calculated from the Lucakova equation for humans and rats (GastroPlus 2018) (Supplementary Table S1). The partition coefficient handles part of the distribution of the CD in compartments based on their lipophilicity, represented by the ratio of the concentration in the tissue/blood at SS. Because of binding in the liver and blood, the partition coefficient showed numbers different than the observed ratios. The parameters for linking the interaction between the ELPC, GIT, liver, and systemic circulation are presented in Supplementary Table S2. The fraction of blood binding and those of proximal and distal absorption from the GIT are presented in Supplementary Table S3. In addition, there are several switches that need to be activated or inactivated, depending on the exposure scenario (Supplementary Table S4).

### Software, algorithms, model code, and statistics

The model was developed using the Advanced Continuous Simulation Language (acslX) version 3.1.5.1 (AEgis Technologies, Huntsville, AL), which allows users to write differential equations and run them as required by the model. The models described with acslX consists of two base files: the first file (extension “.csl”) contains the codes that make up the model; the second file (extension “.m”) contains the model parameters adapted after simulations. Parameter fitting was driven using the relative-error model estimation. Maximization of the log likelihood function was critical for fitting.

### Calibration of the model (and not predictability)

Calibration of the rat and human models was performed using data from published studies and is graphically represented in Supplementary Figs. S1, S2, and S3 for rats and S4 and S5 for humans.

### Predictability assessment

Predictability was assessed using three studies that focused on low and high doses and repetitive doses at different times for rats. For humans, three different simulations of reverse dosimetry assessment were conducted using blood CD concentrations: (1) 38 sequential measurement from workers highly exposed in Hopewell (Adir et al. 1978), (2) those of four different groups of people related to Hopewell (Gr A represents workers revealing illness, Gr B workers without illness, Gr C the family members of workers, and Gr D residents living near the Hopewell plant), for which we used the means of the detectable blood CD measurements for each group (Cannon et al. 1978), and (3) 671 individual measurements from healthy adult males in the general population of the FWI ranging in age from 45 to 88 years (median age 60.6 years) and distributed in quartiles (Emeville et al. 2015).

### Sensitivity analysis

Sensitivity analysis (SA) identifies the ways in which the human model response (here, plasma concentration at SS) changes under the influence of an individual parameter (Easterling et al. 2000). The results are expressed as the magnitude of change for the endpoint of interest. All 40 parameters in the PBPK model were subjected to SA using a daily CD exposure of 1.0 × 10^–3^ µg/kg bw/day for 655 days, corresponding to five blood *T*_1/2_. For the SA, each parameter was varied by ± 5% and compared to the optimised value to determine the influence of small changes in blood concentration using Eq. (1):

1 $$\mathrm{SA}\%=\frac{{\mathrm{Cplasma}}_{\pm 10\%}-{\mathrm{Cplasma}}_{\mathrm{optimized}}}{{\mathrm{Cplasma}}_{\mathrm{optimized}}}\times 100\%.$$

## Results

### Observations from the experiments

#### Assessment and optimization of the model (and not predictability)

The model simulates physiological variables in rats and humans that are within reasonable limits in terms of the dataset. The variables used were largely identical for the physiology of the two species (rats and humans) considered. Several parameters were estimated, including tissue permeability for the adult brain (blood–brain barrier) and the extraction coefficient. The extraction coefficient was optimised for blood and liver concentrations.

#### PBPK model development and calibration for rats

We calibrated the rat PBPK model using three published datasets: (1) a single oral dose of 1 mg CD/kg bw (Bungay et al. 1981), (2) a single oral dose of 40 mg CD/kg bw followed by sequential measurements from day 1 to day 182 for blood, liver, adipose tissue, and faeces, and 3) from day 1 to day 7 for urine (Egle et al. 1978) (Supplementary Figs. S1 to S3). This simulation made it possible to adjust for enterohepatic circulation, GIT excretion from the blood, and the excretion constant in the faeces and urine.

#### PBPK model development and calibration for humans

The objective of this task was to assess the match between the measured concentrations in blood, liver, and adipose tissue over time. A simulation using a sub-chronic exposure scenario assumed the concentration measurements were conducted until SS because workers were exposed at Hopewell plant over a certain period of time until SS was presumably reached (Cohn et al. 1976). Thus, the blood, liver, and adipose tissue concentrations were simulated for 1000 days, corresponding to a plateau with a blood *T*_1/2_ = 131 days (Hallare and Gerriets 2020). The corresponding daily CD dose was 0.19 mg CD/kg bw/day (Supplementary Fig. S4). All three-simulated compartments (whole blood, fat, and liver) precisely reached the measured data point for blood, liver, and adipose tissue (Cohn et al. 1976). These simulations appear to be reasonable and confirm the good predictability of the PBPK model.

### Predictability of the model

#### Rat model

**Fig. 2:**
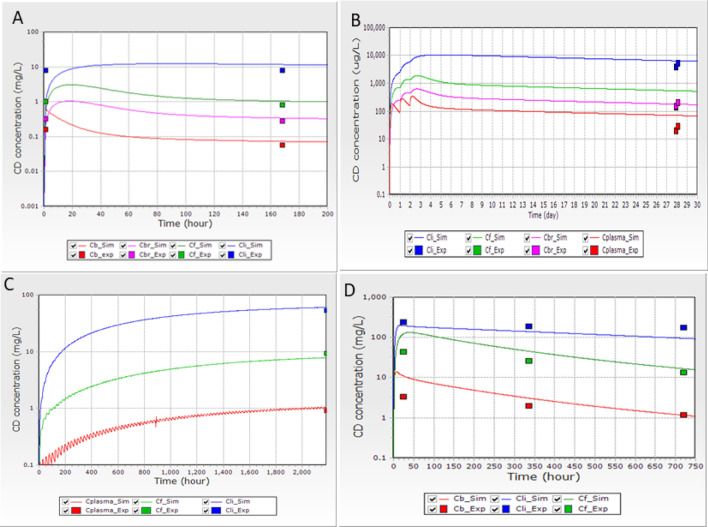
**A** Distribution of CD following a single intravenous exposure to 1 mg CD/kg bw. The experimental data were from Bungay et al. (1981). **B** Distribution of CD following exposure to 0.33 mg of CD/kg bw in the diet for three days. The experimental data were from Richter et al. (1979). **C** Distribution of CD following oral exposure to 5 ppm in the diet for 90 days and sampling 24 h after the last exposure on day 91 (2184 h the beginning of the treatment). The experimental data were from Linder et al. (1983). **D** Distribution of CD in male rats after a single oral dose of 40 mg/kg bw. The experimental data were from Belfiore et al. (2007). _Sim is the simulation profile and _Exp is the experimental data measured. The CD concentration (mg/L) or (µg/L) is shown on the Y axes and the hours or days on the X axes. *Cb* CD in the blood, *Cbr* CD in the brain, *CF* CD in the adipose tissue, *CLi* CD in the liver

We assessed the predictability of the PBPK model using four studies (see Fig. 2A–D). The first (Fig. 2A) represented a single intravenous exposure dose of 1 mg CD/kg bw in rats (Bungay et al. 1981). The second (Fig. 2B) corresponded to an oral exposure of 0.33 mg CD/kg of bw/day for three consecutive days followed by measuring CD concentrations in the blood, liver, and adipose tissue for 25 days post-exposure. The third (Fig. 2C) represented a sub-chronic exposure to CD in a diet at a dose of 5 ppm, corresponding to 0.125 mg CD/kg bw/day, (based on 10 g of food per 100 g of animal bw, for animals with 250 g of bw) for 90 days, followed by sacrifice of the animals and sampling to measure blood, liver, and adipose tissue 24 h after the last exposure. The fourth study (Fig. 2D) corresponded to a single oral exposure of 40 mg CD/kg bw, followed by sacrifice of the animals at 24, 336, and 720 h post-exposure and measurement of CD concentrations in the blood, liver, and adipose tissue. All simulations adequately reproduced the experimental values (For more simulations, see Supplementary Figs. S5 to S17).

#### Human model

**Fig. 3:**
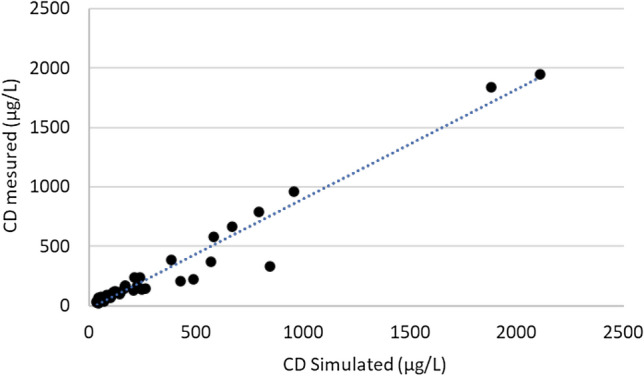
Comparison between simulated blood concentrations (*n* = 38) and sequential measurements of CD in humans (Adir et al. 1978). A Spearman’s nonparametric test showed a Rho correlation of 0.96 (*p* < 0.001)

The human PBPK model simulation was compared to 38 measured concentrations of CD in the blood of 12 CD workers at Hopewell plant (Fig. 3). Each worker had three or four sequential data points. CD blood concentrations ranged between 120 and 2109 µg/L (Adir et al. 1978). A Spearman’s nonparametric test was used to compare the experimental to the simulation values and showed a Rho (*ρ*) correlation of 0.960 (*p* < 0.001).

**Fig. 4:**
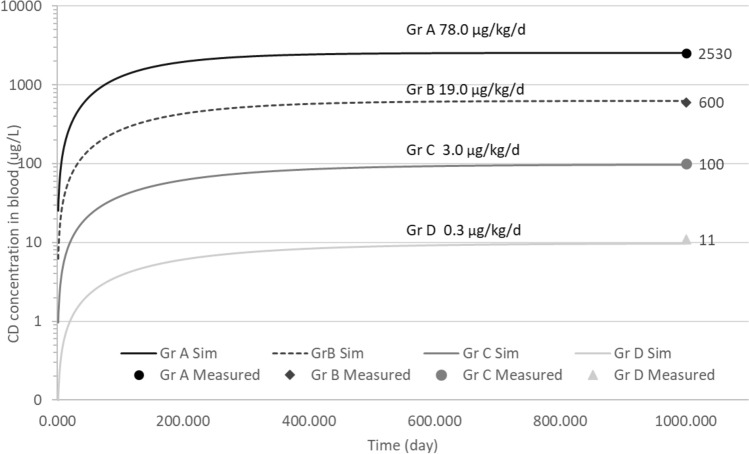
Profile concentrations of CD in the blood (expressed in µg/L) after repetitive exposure to different daily doses (in mg/kg bw/day). Each lines represents different a simulation; dots represent the mean of the measured concentrations in blood for the different groups. Gr A represents affected workers, Gr B unaffected workers, Gr C the family members of a worker, and Gr D Hopewell residents (Cannon et al. 1978)

A second scenario reproduced the CD blood concentration at SS of four groups of exposed people at Hopewell (Cannon et al. 1978). The simulation spanned affected plant workers (Gr A) to Hopewell residents who were not directly exposed to CD at the plant (Gr D) (Fig. 4). Evaluation of the daily exposure scenario made it possible to determine the external mean concentrations to which the four groups were exposed.

### Prediction of external doses for the French West Indies population based on blood concentrations

**Fig. 5:**
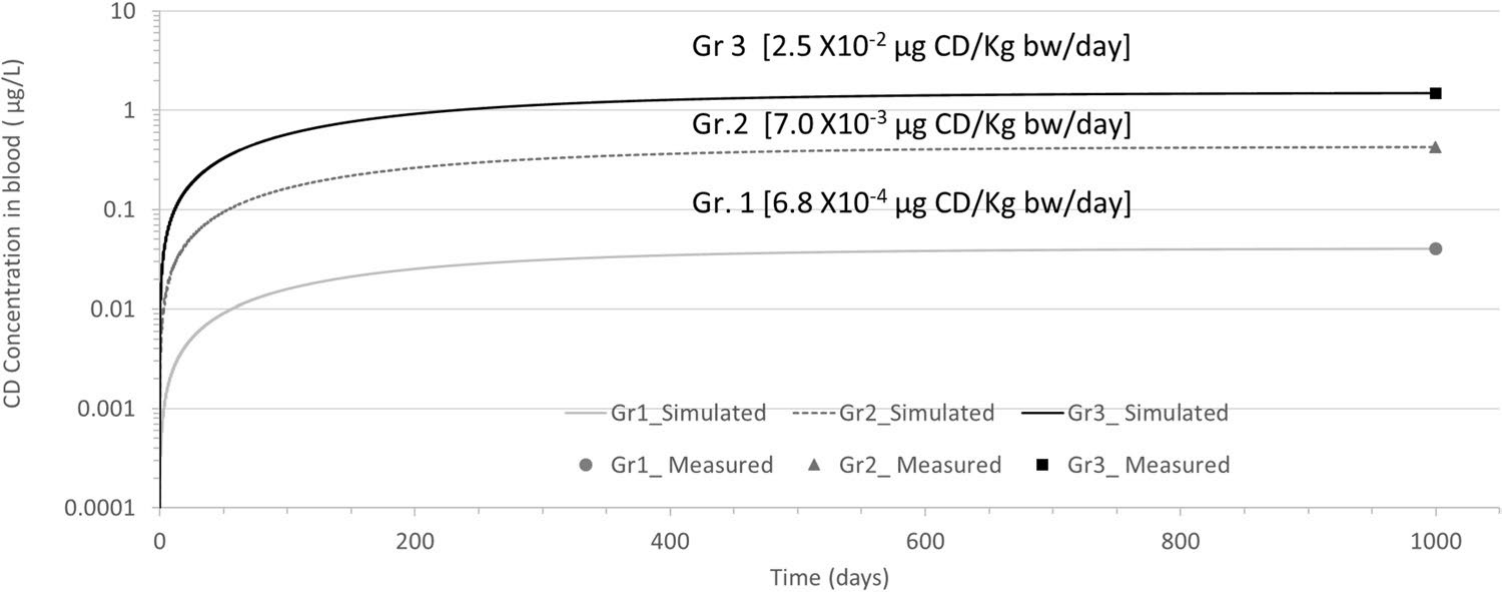
Profile simulation reproducing the daily exposure of 671 adult healthy males from FWI. Three groups of blood concentrations were measured: Gr. 1 (*n* = 166), Gr. 2 (*n* = 337), and Gr. 3 (*n* = 168). Lines represent the simulation. Dots represent the blood concentration for each group for 1000 days. The measurements at 1000 days are arbitrary and correspond to the SS in blood based on a half-life of 131 days

We conducted a simulation using the oral daily dose expressed in µg of CD/kg bw/day and compared it to a population of healthy adult men living in the FWI as a function of the percentile of the plasma CD concentration (internal dose). Among the 671 men, 166 had blood CD concentrations below the 25th percentile (Gr. 1 [< 0.18 µg/L, median 0.04 µg/L]), 337 between the 25th and 75th percentiles (Gr. 2 [0.18 to 0.86 µg/L, median of 0.42 µg/L]), and 168 above the 75th percentile (Gr. 3 [0.86 to 49.12 µg/L, median 1.48 µg/L]) (Emeville et al. 2015). Values below the limit of detection (0.06 µg/L) were imputed as the limit of detection/square root of 2 (Hornung and Reed 1990). The simulated external doses using the median values were 0.00068, 0.007, and 0.025 µg CD/kg bw/day, respectively (Fig. 5).

### Model sensitivity analysis (SA)

A SA of the human PBPK model was conducted for all 40 parameters (Supplementary Fig. S18). The parameters retained were those for which a change by ± 5% of the parameter values influenced the output blood concentration by more than ± 0.2% following chronic oral exposure of 0.001 µg/kg bw/day for 655 days. The most sensitive parameters observed were BIND (the fraction of CD bound to blood proteins), KA (absorption fraction in the GIT), KBILE (the metabolism fraction), KST (the unabsorbed fraction in the GIT), LIBMAXCD1 (the concentration of protein CDBP in the liver), and PF (the partition coefficient between adipose tissue and the blood).

## Discussion

In the FWI, the pesticide CD was used in banana farming from 1973 to 1993, resulting in permanently polluted soil and waterways. Recent epidemiological studies identified health effects resulting from environmental exposure (Legoff et al. 2021b; Multigner et al. 2016). The chronic external TLV_external_ for CD recently reassessed (Anses 2021) highlighted the difficulty of performing such assessments without a human PBPK model. The objective of this study was to develop a mathematical PBPK model in rats using data in the literature and then extrapolate it to a human model to support health regulatory agencies.

Rats and humans share similar pharmacokinetic properties, except for metabolism, as rats have a limited hepatic CD reductase capacity to convert CD into its reduced form CD-OH (Houston et al. 1981; Molowa et al. 1986). In terms of absorption, distribution, and elimination, most CD is found in the rats’ faeces (Houston et al. 1981), whereas CD and CD-OH are found in human faeces (Fariss et al. 1978). The enterohepatic circulation and blood (Belfiore et al. 2007; Skalsky et al. 1979b) and hepatic binding (Guzelian et al. 1981) provide a plausible explanation for the long half-life of CD described for rats and humans (Boylan et al. 1978; Guzelian 1981). Only parent CD is present in the blood of both species (Fariss et al. 1980). In humans, the CD-OH found in faeces comes solely from bile (Guzelian 1981). Only negligible fractions of CD-OH have been detected in the urine and plasma for both rats and humans (Fariss et al. 1978, 1980). Reconversion of CD-O-G and CD-OH to CD from GIT is mathematically described in this human PBPK model (Cohn et al. 1978; Scheline 1973).

The rat PBPK model was optimised with experimental measurements, such as a single oral low dose of 1 mg CD/kg bw (Bungay et al. 1981) and a single oral high dose of 40 mg CD/kg bw in rats, followed by sequential sampling (Egle et al. 1978). After optimization, the simulation for various exposure scenarios was relatively accurate, even when it involved a single exposure (Belfiore et al. 2007; Bungay et al. 1981) or repetitive exposure in the diet or by gavage (Linder et al. 1983; Richter et al. 1979). Addition simulations showing relatively good prediction are presented in Supplementary Figs. S5 to S17. Part of the old rat experimental dataset involved ^14^C labelled CD, making it impossible to distinguish the metabolites from the parent compound. This may explain part of the discrepancy between the observed and simulated profiles. Overall, we consider our rat model to be adequate for risk assessments and extrapolation to humans. For all rat models described in the supplementary materials, that of Belfiore et al. (2007) provided graphs. This allowed us to compare the present model with the Belfiore PBPK model. The Belfiore model simulated their own data and that of Egle et al. (1978). Our rat PBPK model resulted in a better prediction than that of Belfiore et al. (2007) when simulating data from Egle et al. (1978) but in a similar prediction with data generated by Belfiore et al. (2007) (Fig. 2d).

Data for the pharmacokinetic mechanisms in the literature related to exposure measurements for humans are limited. Thus, we used the same coding template as that used for rats to improve confidence in the model structure. For the extrapolation, we studied and optimised the parameters based on the Hopewell observations. We assessed the predictive quality of the model by simulating workers highly exposed to CD at the Hopewell plant (Cohn et al. 1976; Taylor et al. 1978). We simulated a daily exposure for 1000 days, corresponding to seven elimination *T*_1/2_ (131 days) and estimated the mean concentration at SS for blood, adipose tissue, and liver and then compared it the mean tissue concentrations from the literature (Cohn et al. 1976; Taylor et al. 1978). This simulation resulted in a good prediction using the reverse dosimetry approach, resulting in an estimated external daily dose of 0.19 mg CD/kg bw/day. Overall, these simulations provide crucial information to enhance our knowledge of the enterohepatic cycle, which is a transit parameter from the blood to the GIT lumen. As we anticipated, the human PBPK model based on the rat PBPK structure accurately predicted the results for three tissues (adipose tissue, blood, and liver concentration) of interest (Supplementary Fig. S5).

Concerning the human PBPK model, we performed two exercises with the same strategy using reverse dosimetry: (1) four groups of people exposed to CD at Hopewell at different exposure levels (from affected workers to Hopewell residents) (Fig. 4) (Cannon et al. 1978) and (2) an adult male population in FWI (Fig. 5) (Emeville et al. 2015). The simulations of external doses for the various groups related to Hopewell suggested that the resident population (Gr D, Fig. 4) was exposed to 0.3 µg CD/kg bw/day, which was 260 times lower than the exposure of the poisoned workers of 78 µg CD/kg bw/day (Gr A Fig. 4). Concerning the FWI population, the median external dose of Gr. 3 (0.025 µg/kg bw/day) and Gr.1 (0.00068 µg/kg bw/day) showed a gap ≈ 37 times between the two groups (Fig. 5), suggesting a large diversity in the exposure profiles of this population. No comparison was possible between the Hopewell and FWI population, because the first dataset considered only detectable CD values and the second all values. We can only mention that the difference was of several orders of magnitude between these two datasets.

Emeville et al. (2015) reported a significant increased risk of prostate cancer among subjects with blood CD concentrations > 1.03 µg/L. The corresponding simulated threshold external oral dose was 0.0176 µg CD/kg bw/day. In another epidemiological study, Kadhel et al. (2014) reported a significantly increased risk of preterm birth for pregnant women with blood CD concentrations > 0.52 µg/L. Assuming a negligible impact of pregnancy on CD blood concentration, the simulated external dose corresponds to 0.009 µg CD/kg bw/day. This suggests that an external CD concentration below 0.009 or 0.0176 µg/kg bw/day may have no effect on humans. These figures appear to be lower than the CD TLV_external_ (0.17 µg/kg bw/day) recommended by the ANSES using the Belfiore rat PBPK model (Anses 2021). This discrepancy can be explained by differences in the pharmacokinetics and pharmacodynamics between the two species (including enterohepatic recirculation, distal GIT secretion, and biliary elimination).

The sensitivity analysis (SA) for the human model was performed by modifying optimized parameters by ± 5%. Such modification of only six among the 40 parameters modified the blood CD concentrations by more than ± 0.2%. This suggests that all the other parameters do not have an important impact, even if there is uncertainty in the accuracy. There are data for BIND and KBILE to support the values we obtained. Overall, the literature captured the pharmacokinetics of these parameters relatively well, which ensured our confidence in using them. We believe that these rat and human models simulate the observations from the literature relatively well.

The human CD PBPK model has several strengths. It can model epidemiological data and directly estimate the mean dose of exposure by reverse dosimetry without an interspecies and intra-species uncertainty factor. This model also describes enterohepatic circulation and the reduction of CD to CD-OH. The limitations of this human PBPK model are related to certain pharmacodynamic components for which our knowledge is still insufficient: the importance of the lymphatic circulation for CD, the liver CDBP, how the CDBP influences CD storage in the liver, and how reabsorption and non-biliary excretion appear in the GIT. Despite these limitations, the present human PBPK model generated predictions that were relatively accurate and in accordance with several simulations of studies in rats (Supplementary Figs S5–S18). In addition, the measured blood concentrations from epidemiological studies improve the confidence in the human PBPK model. This human PBPK model will be important in correlating the doses of external exposure to health effects based on blood concentrations and in supporting governmental agencies in updating the chronic TLV based on human data.

## Conclusion

This is the first human PBPK model applied to CD. Such a model can support health regulatory agencies in their efforts to help FWI populations currently exposed to CD. This model was designed, optimized, and assessed using all the data available in the literature for rats. We also performed a comparison with available human data. This human PBPK model is a good predictive tool and can be used to estimate external CD exposure dose scenarios based on internal CD blood concentrations.

## Supplementary Information

Below is the link to the electronic supplementary material.

Supplementary file1 (DOCX 661 KB)

## Abbreviations

acslX: Advanced continuous simulation language
ANSES: French Agency for Food, Environmental and Occupational Health & Safety (Agence Nationale de Sécurité Sanitaire de l'Alimentation, de l'Environnement et du Travail)
CAS: Chemical abstracts service
CD: Chlordecone
CDBP I and II: Chlordecone-binding proteins I and II
CD-OH: Chlordecone alcohol or chlordecol
CD-OH-G: Chlordecone alcohol glucuronide
d: Day
FWI: French West Indies
GIT: Gastrointestinal tract
HED: Human equivalent dose
PBPK: Physiologically based pharmacokinetic
RE: Rest of the body
SA: Sensitivity analysis
SS: Steady state
*T*_1/2_: Half-life

## References

[CR1] Adir J, Caplan YH, Thompson BC (1978). Kepone serum half-life in humans. Life Sci.

[CR2] Andersen ME, Clewell II (1987). Physiologically based pharmacokinetics and the risk assessment process for methylene chloride. Toxicol Appl Pharmacol.

[CR3] Anses (2017) Exposition des consommateurs des Antilles au chlordécone, résultats de l'étude Kannari Rapport d’expertise collective. Anses, Maisons-Alfort, p 202

[CR4] Anses (2021) Valeurs sanitaires de référence: Le chlordecone. Anses, Maisons-Alfort, p 154

[CR5] ATSDR (2020) Toxicological profile for mirex and chlordecone. p 332 37075159

[CR6] Belfiore CJ, Yang RSH, Chubb LS, Lohitnavy M, Lohitnavy OS, Andersen ME (2007). Hepatic sequestration of chlordecone and hexafluoroacetone evaluated by pharmacokinetic modeling. Toxicology.

[CR7] Bocquene G, Franco A (2005). Pesticide contamination of the coastline of Martinique. Mar Pollut Bull.

[CR8] Boucher O, Simard MN, Muckle G (2013). Exposure to an organochlorine pesticide (chlordecone) and development of 18-month-old infants. Neurotoxicology.

[CR9] Boylan JJ, Egle JL, Guzelian PS (1978). Cholestyramine: use as a new therapeutic approach for chlordecone (kepone) poisoning. Science.

[CR10] Boylan JJ, Cohn WJ, Egle JL, Blanke RV, Guzelian PS (1979). Excretion of chlordecone by the gastrointestinal tract: evidence for a nonbiliary mechanism. Clin Pharmacol Ther.

[CR11] Boylan JJ, Egle, Jr., Guzelian PS (1977) Stimulation of chlrodecone (CD) (Kepone) excretion by Chlolestyramine (Cs) in rats. vol 19, p 210

[CR12] Bungay PM, Dedrick RL, Matthews HB (1981). Enteric transport of chlordecone (Kepone) in the rat. J Pharmacokinet Biopharm.

[CR13] Bungay PM, Dedrick RL, Matthews HB (1980) Pharmacokinetics of Environmental contaminants. Dynamics, exposure and hazard assessment of toxic chemicals, pp 369–377

[CR14] Cabidoche YMAR, Cattan P, Clermont-Dauphin C, Massat F, Sansoulet J (2009). Long-term pollution by chlordecone of tropical volcanic soils in the French West Indies: a simple leaching model accounts for current residue. Environ Pollut.

[CR15] Cannon SB, Veazey JM, Jackson RS (1978). Epidemic kepone poisoning in chemical workers. Am J Epidemiol.

[CR16] Carpenter HM, Curtis LR (1991). Low dose chlordecone pretreatment altered cholesterol disposition without induction of cytochrome P-450. Drug Metab Dispos.

[CR17] Cohn WJ, Boylan JJ, Blanke RV, Fariss MW, Howell JR, Guzelian PS (1978). Treatment of chlordecone (Kepone) toxicity with cholestyramine. Results of a controlled clinical trial. N Engl J Med.

[CR18] Cohn WJ, Blanke RV, Griffith FD, Guzelian PS (1976) Distribution and excretion of Kepone (KP) in humans. vol 71, p 901

[CR19] De Winne D (1979). Influence of blood flow on intestinal absorption of drugs and nutriments. Pharm Ther.

[CR20] Dereumeaux C, Saoudi A, Guldner L (2019). Chlordecone and organochlorine compound levels in the French West Indies population in 2013–2014. Environ Sci Pollut Res Int.

[CR21] Dubuisson C, Heraud F, Leblanc JC (2007). Impact of subsistence production on the management options to reduce the food exposure of the Martinican population to Chlordecone. Regul Toxicol Pharmacol.

[CR22] Easterling MR, Evans MV, Kenyon EM (2000). Comparative analysis of software for physiological based pharmacokinetic modeling: simulation, optimization and sensibility analysis. Toxicol Mech Methods.

[CR23] Egle JL, Fernandez JB, Guzelian PS, Borzelleca JF (1978). Distribution and excretion of chlordecone (Kepone) in the rat. Drug Metab Dispos.

[CR24] Emeville E, Giusti A, Coumoul X, Thome JP, Blanchet P, Multigner L (2015). Associations of plasma concentrations of dichlorodiphenyldichloroethylene and polychlorinated biphenyls with prostate cancer: a case-control study in Guadeloupe (French West Indies). Environ Health Perspect.

[CR25] Emond C, DeVito M, Warner M, Eskenazi B, Mocarelli P, Birnbaum LS (2016). An assessment of dioxin exposure across gestation and lactation using a PBPK model and new data from Seveso. Environ Int.

[CR26] Fabacher DL, Hodgson E (1976). Induction of hepatic mixed-function oxidase enzymes in adult and neonatal mice by kepone and mirex. Toxicol Appl Pharmacol.

[CR27] Fariss MW, Blanke RV, Saady JJ, Guzelian PS (1980). Demonstration of major metabolic pathways for chlordecone (kepone) in humans. Drug Metab Dispos.

[CR28] Fariss MW, Blanke RV, Boylan JJ, King ST, Guzelian PS (1978) Reductive biotransformation of chlordecone in man and rat. vol 45, p 337

[CR29] GastroPlus (2018) GastroPlus Simulation software for drug discovery and development (version 9.6). Simulation Plus, p 748

[CR30] Gely-Pernot A, Hao C, Legoff L (2018). Gestational exposure to chlordecone promotes transgenerational changes in the murine reproductive system of males. Sci Rep.

[CR31] Gilroy DJ, Carpenter HM, Curtis LR (1994). Chlordecone pretreatment alters [14C]chlordecone and [14C]cholesterol transport kinetics in the perfused rat liver. Fundam Appl Toxicol.

[CR32] Guldner L, Multigner L, Heraud F (2010). Pesticide exposure of pregnant women in Guadeloupe: ability of a food frequency questionnaire to estimate blood concentration of chlordecone. Environ Res.

[CR33] Guzelian PS (1981). Therapeutic approaches for chlordecone poisoning in humans. J Toxicol Environ Health.

[CR34] Guzelian PS (1982). Chlordecone poisoning: a case study in approaches for detoxification of humans exposed to environmental chemicals. Drug Metab Rev.

[CR35] Guzelian PS (1982). Comparative toxicology of chlordecone (Kepone) in humans and experimental animals. Annu Rev Pharmacol Toxicol.

[CR36] Guzelian PS, Mutter L, Fariss MW, Blanke RV (1981) Metabolism and biliary excretion of Chlordecone (Kepone) in humans. In: Khan MAQ, Stanton RH (eds) Toxicology of halogenated hydrocarbons Health and Ecological effects. Pergamon Press Inc., pp 315–325

[CR37] Hallare J, Gerriets V (2020) Half life StatPearls. Treasure Island 32119385

[CR38] Hornung RW, Reed LD (1990). Estimation of average concentration in the presence of nondetectable values. Appl Occup Environ Hyg.

[CR39] Houston TE, Mutter LC, Blanke RV, Guzelian PS (1981). Chlordecone alcohol formation in the Mongolian gerbil (*Meriones unguiculatus*): a model for human metabolism of chlordecone (kepone). Fundam Appl Toxicol.

[CR40] Kadhel P, Monfort C, Costet N (2014). Chlordecone exposure, length of gestation, and risk of preterm birth. Am J Epidemiol.

[CR41] Kadhel P (2008) Pesticides in the Antilles, impact on the function of reproduction [in French]. PhD thesis. Université des Antilles et de la Guyane, Guadeloupe, French West Indies

[CR42] Krishnan K, Andersen M, Hayes AW (2008). Physiologically based pharmacokinetic and toxicokinetic models. Principles and methods of toxicology.

[CR43] Larson PS, Egle JL, Hennigar GR, Lane RW, Borzelleca JF (1979). Acute, subchronic, and chronic toxicity of chlordecone. Toxicol Appl Pharmacol.

[CR44] Legoff L, Dali O, D'Cruz SC (2019). Ovarian dysfunction following prenatal exposure to an insecticide, chlordecone, associates with altered epigenetic features. Epigenetics Chromatin.

[CR45] Legoff L, D'Cruz SC, Bouchekhchoukha K (2021). In utero exposure to chlordecone affects histone modifications and activates LINE-1 in cord blood. Life Sci Alliance.

[CR46] Legoff L, D'Cruz SC, Lebosq M (2021). Developmental exposure to chlordecone induces transgenerational effects in somatic prostate tissue which are associated with epigenetic histone trimethylation changes. Environ Int.

[CR47] Linder RE, Scotti TM, McElroy WK, Laskey JW, Strader LF, Powell K (1983). Spermotoxicity and tissue accumulation of chlordecone (Kepone) in male rats. J Toxicol Environ Health.

[CR48] Luecke RH, Pearce BA, Wosilait WD, Slikker W, Young JF (2007). Postnatal growth considerations for PBPK modeling. J Toxicol Environ Health A.

[CR49] Matthews HB (1979). Excretion of insecticides. Pharmacol Ther B.

[CR50] Molowa DT, Shayne AG, Guzelian PS (1986). Purification and characterization of chlordecone reductase from human liver. J Biol Chem.

[CR51] Multigner L, Ndong JR, Giusti A (2010). Chlordecone exposure and risk of prostate cancer. J Clin Oncol.

[CR52] Multigner L, Kadhel P, Rouget F, Blanchet P, Cordier S (2016). Chlordecone exposure and adverse effects in French West Indies populations. Environ Sci Pollut Res Int.

[CR53] Ramsey JC, Andersen ME (1984). A physiologically based description of the inhalation pharmacokinetics of styrene in rat and human. Toxicol Appl Pharmacol.

[CR54] Reichl D (1994). Extravascular circulation of lipoproteins: their role in reverse transport of cholesterol. Atherosclerosis.

[CR55] Richter E, Lay JP, Klein W, Korte F (1979). Enhanced elimination of kepone-14C in rats fed liquid paraffin. J Agric Food Chem.

[CR56] Scheline RR (1973). Metabolism of foreign compounds by gastrointestinal microorganisms. Pharmacol Rev.

[CR57] Seurin S, Rouget F, Reninger JC (2012). Dietary exposure of 18-month-old Guadeloupian toddlers to chlordecone. Regul Toxicol Pharmacol.

[CR58] Skalsky HL, Fariss MW, Blanke RV, Guzelian PS (1979). The role of plasma protein in the transport and distribution of chlordecone (KEPONE) and other polyhalogenated hydrocarbons. Ann N Y Acad Sci.

[CR59] Skalsky HL, Fariss MW, Blanke RV, Guzelian PS (1979). The role of plasma proteins in the transport and distribution of chlordecone (Kepone) and other polyhalogenated hydrocarbons. Ann N Y Acad Sci.

[CR60] Soine PJ, Blanke RV, Guzelian PS, Schwartz CC (1982). Preferential binding of chlordecone to the protein and high density lipoprotein fractions of plasma from humans and other species. J Toxicol Environ Health.

[CR61] Soine PJ, Blanke RV, Schwartz CC (1983). Chlordecone metabolism in the pig. Toxicollett.

[CR62] Soine PJ, Blanke RV, Schwartz CC (1984). Isolation of chlordecone binding proteins from pig liver cytosol. J Toxicol Environ Health.

[CR63] Taylor JR, Selhorst JB, Houff SA, Martinez AJ (1978). Chlordecone intoxication in man. I. Clinical observations. Neurology.

[CR64] USEPA (2010) EPA's reanalysis of key issues related to dioxin toxicity and response to NAS comments vol EPA/600/R-10/038A, pp 1–1849

