# Supplementary material for: Chlordecone: development of a physiologically based pharmacokinetic tool to support human health risks assessments
Source: Arch Toxicol. 2022 Feb 5;96(4):1009–19. doi: 10.1007/s00204-022-03231-3 (PMC8921106; doi:10.1007/s00204-022-03231-3)
Supplement: Supplementary file 1 — Supplementary file1 (DOCX 661 KB) [file 204_2022_3231_MOESM1_ESM.docx]

### **SUPPLEMENTAL MATERIAL**

**Chlordecone: Development of a physiologically based pharmacokinetic tool to support assessments of health risks (by Claude Emond and Luc Multigner)**

###

### **Table of Contents**

| 1 | Previous PBPK model developed for rodents | 1 |
| --- | --- | --- |
| 2 | Supplementary tables (Tables S1 to S4) | 3 |
|  | Supplementary Figures | 6 |
|  | PBPK model development and calibration for rat (Figures S1 to S3) | 6 |
|  | PBPK model development and calibration for human (Figures S4) | 8 |
|  | Predictability of the rat model (Figures S5 to S17) | 9 |
| 3 | Sensitivity analysis of human model (Figure S18) | 15 |
| 4 | Parameters and variables symbols used in the models (units) | 15 |
| 5 | Equations used for human model and their units | 16 |
| 6 | References | 21 |

### **1. Previous PBPK model developed for rodents**

Five PBPK models relating to chlordecone have been published. All describe rodent pharmacokinetics. The first was published in 1979 and included six compartments (Bungay et al. 1979). This model was developed for halogenated hydrocarbons and adapted for chlordecone. Indeed, the model structure was used for both hexachlorobiphenyl and chlordecone. The model does not discuss or consider the enterohepatic cycle, involved in the metabolism of chlordecone. These same authors later used this model structure and increased the number of compartments from six to 10 (Bungay et al. 1981). According to the authors’ description, each digestive compartment has the capacity to absorb chlordecone and passively eliminate it from the bloodstream. In 1989, Shah et al. published a PBPK model describing the dermal absorption of chlordecone with five compartments (Shah et al. 1989). In this PBPK model for dermal exposure, chlordecone was introduced directly into the blood, even though the model has a skin compartment. (Shah et al. 1989; Shah et al. 1987). This is a simplification of the absorption process and bypasses the skin diffusion rate, thus reducing the physiological plausibility of the model. No graph to assess the prediction of the model was presented. In 1998, a four-compartment model was developed to describe the percutaneous absorption of chlordecone in prepubertal and adult rodents (Heatherington et al. 1998). This model describes the transfer of chlordecone into three different strata of the skin as three sub-compartments. According to the authors, this model requires further study to better characterize cutaneous transfer. They also stated that elements concerning the blood-binding characteristics of chlordecone and the uptake of bound chlordecone by the liver would also need to be added. The authors mentioned that they did not include enterohepatic circulation because they believed there to be no evidence of recirculation and the intestine was not a tissue of interest for this PBPK model (Heatherington et al. 1998). Belfiore et al. (2007) developed a model with four compartments (Belfiore et al. 2007). The model describes oral exposure and hepatic elimination. Although this model can be extrapolated to humans, there is uncertainty about the parameters used and certain important physiological descriptions are missing. In summary, the PBPK models previously reported in the literature do not describe simulations of lifetime exposure or the enterohepatic cycle, which are important PK components. None of the published models in rodents allow them to be extrapolated to humans without structural modification. One of our objectives in this study was to produce a typical rodent PBPK model and then use its structure for human extrapolation.

**2. Supplementary Tables**

**Supplementary Table S1: Constants and parameters used in the model for rats and humans**

| **Parameters** | **Values** | |
| --- | --- | --- |
|  | **Rat** | **Human** |
| ***Cardiac output and pulmonary output (L/min/Kg) or (L/d/kg)*** | | |
| QCC (Cardiac output) | 0.235 ^a^ (L/min/kg) | 338.4 ^a^ (L/d/kg) |
| QPC (Alveolar pulmonary output) | 0.330 ^a^ (L/min/kg) | 475.2 ^a^ (L/d/kg) |
| ***Fraction of blood in compartment (unitless)*** | | |
| QBR0 (Brain) | Variable ^b^ | Variable ^b^ |
| QF0 (Adipose tissue) | Variable ^b^ | Variable ^b^ |
| QLI0 (Liver) | Variable ^b^ | Variable ^b^ |
| QLUNG0 (Lung) | Variable ^b^ | Variable ^b^ |
| QRB0 (RE) | Variable ^b^ | Variable ^b^ |
| QSK0 (Skin) | Variable ^b^ | Variable ^b^ |
| ***Fraction volume of compartment (unitless)*** | | |
| VB0 (Blood) | Variable ^b^ | Variable ^b^ |
| VBR0 (Brain) | Variable ^b^ | Variable ^b^ |
| VF0 (Adipose tissue) | Variable ^b^ | Variable ^b^ |
| VLI0 (Liver) | Variable ^b^ | Variable ^b^ |
| VRB0 (RE) | Variable ^b^ | Variable ^b^ |
| VSK0 (Skin) | Variable ^b^ | Variable ^b^ |
| ***Fraction of tissue blood volume compartment (unitless)*** | | |
| VBRB0 (Brain) | 0.266 ^c^ | 0.0096 ^c^ |
| VFB0 (Adipose tissue) | 0.266 ^d^ | 0.266 ^d^ |
| VLIB0 (Liver) | 0.266 ^d^ | 0.266 ^d^ |
| VSKB0 (Skin) | 0.266 ^d^ | 0.036 ^d^ |
| ***Fraction of permeability limited between tissue blood and cellular matrix (unitless)*** | | |
| PABRF (Brain) | 0.0096 ^c^ | 0.0096 ^c^ |
| PAFF (Adipose tissue) | 0.12 ^c^ | 0.1285 ^c^ |
| PALIF (Liver) | 0.12 ^c^ | 0.59 ^c^ |
| PASKF (Skin) | 0.01 ^c^ | 0.01 ^c^ |
| ***Partition coefficient (L of blood/L of tissue) or (L of blood/L of air) (unitless)*** | | |
| Pb (Blood/air) | 1.0×10^+10^ ^c^ | 1,0×10^+10^ ^c^ |
| Plu (Lung/blood) | 0.94 | 0.94 ^e^ |
| Pbr (Brain/blood) | 22.78 ^e^ | 11.13 ^e^ |
| Pf (Adipose tissue/blood | 70 ^e^ | 25.75 ^e^ |
| Pli (Liver/blood) | 9.5 ^e^ | 6.98 ^e^ |
| Prb (RE/blood) | 8.5 ^e^ | 4.27 ^e^ |
| Psk (Skin/blood) | 12.12 ^e^ | 5.22 ^e^ |

^a^ (Krishnan and Andersen 2008); ^b^(Luecke et al. 2007); ^c^ Optimised; ^d^ (Emond et al. 2004); ^e^ (GastroPlus 2018)

**Supplementary Table S2: Constants describing the interaction between the liver, ELPLC, GIT, and Systemic circulation**

| **Parameters** | **Values** | |
| --- | --- | --- |
|  | **Rat** | **Human** |
| ***Concentration of protein binding in liver*** | | |
| LIBMAXCD1 (CD1 in liver) | 4.5×10^-1^ ^a^ mmol/L | 0.21 (mmol/L) ^a^ |
| LIBMAXCD2 (CD2 in liver) | 2.16×10^-1^ ^a^ mmol/L | 0.1 (mmol/L) ^a^ |
| ***Protein affinity constant in liver*** | | |
| KDLI1 (CD1 Cte) | 5.9×10^-4^ mmol/L ^a^ | 5.9×10^-4^ (mmol/L) ^a^ |
| KDLI2 (CD2 Cte) | 3.1×10^-2^ mmol/L ^a^ | 3.1×10^-2^ (mmol/L) ^a^ |
| ***Liver and GIT interaction*** | | |
| KBILEAM (Elimination CD-OH into bile) | 0.1 ^a^ (h-1) ^a^ | 15 (d^-1^) ^a^ |
| KELMET (Metabolic Cte from CD to CD-OH in liver) | 1.0×10^-4^ ^a^ (h^-1^) ^a^ | 0.05 (d^-1^) ^a^ |
| KBILE (Elimination of CD into bile) | 0.27 ^a^ (h^-1^) ^a^ | 3.7 (d^-1^) ^a^ |
| KCONVAMCD (Reconversion of CD-OH into CD in GIT) | 1.0 (h^-1^) ^a^ | 1.0×10^-4^ (d^-1^) ^a^ |
| CFLLI0 (Initial concentration of CD in liver) | 0 | 0 |
| ***Fraction of the amount in the GIT in the portal circulation*** | | |
| KA (Proximal oral absorption Cte) | 0.3 ^a^ (h^-1^) ^a^ | 7.2 (d^-1^) ^a^ |
| KA1 (Distal oral absorption Cte) | 0.3 ^a^ (h^-1^) ^a^ | 1.0×10^-4^ (d^-1^) ^a^ |
| KST (Oral proximal no absorption constant) | 0.045 ^a^ (h^-1^) ^a^ | 1.08 (d^-1^) ^a^ |
| KST1 (Oral distal no absorption constant) | 1.5 (h^-1^) ^a^ | 12 (d^-1^) ^a^ |
| KST1AM (Oral distal none absorption constant for metabolite) | 0.0 (h-1) ^a^ | 2 (d^-1^) ^a^ |
| KBGITEXP (Constant transmural CD in GIT) | 1.01×10^-2^ (L/h) ^a^ | 1.01×10^-3^ (L/d) ^a^ |
| ***ELPLC*** | | |
| KLIPO_I (CD in ELPLC from blood compartment [blood]) | 3 (h^-1^) ^a^ | 72 (d^-1^) ^a^ |
| KLIPO_O (CD leaving ELPLC to the blood compartment) | 30 (h^-1^) ^a^ | 720 (d^-1^) ^a^ |
| KLIPO_OLI (CD leaving ELPLC to the liver compartment) | 3 (h^-1^) ^a^ | 72 (d^-1^) ^a^ |

^a^ Optimised

**Supplementary Table S3: Miscellaneous constant used in the PBPK model for rats and humans**

| **Parameters** | **Values** | |
| --- | --- | --- |
|  | **Rat** | **Human** |
| ***Protein binding in blood (Albumin, HDL, LDL) (unitless)*** | | |
| BIND (Fraction of total blood) | 0.7 ^a^ | 0.7 ^a^ |
| ***Fraction of absorption from the proximal GIT in the portal vein or ELPLC (unitless)*** | | |
| A (Absorption fraction of Ka of proximal absorption) | 0.3 ^a^ | 0.3 |
| A1 (Absorption fraction of Ka1 distal absorption) | 0.9 ^a^ | 0.3 |
| ***Absorption or elimination constant (h^-1^) or switch (0 or 1****)* | | |
| CLURI (Urinary elimination clearance) | 2.0×10^-4^ (L/h) ^b^ | 4.80×10^-3^ (L/d) ^a^ |

^a^ Optimised

**Supplementary Table S4: switch used in the models**

| **Parameters** | **Values** | | |
| --- | --- | --- | --- |
|  | **Rat** | | **Human** |
| ***Liver and GIT interaction*** | | | |
| BILE_SWITCH (activ. switch bile excretion of CD) | 0 or 1 | 0 or 1 | |
| BILE_SWITCHAM (Act. switch bile excreted CD-OH) | 0 or 1 | 0 or 1 | |
| SWITCH_CONV (activate conversion of CD-OH TO CD in GIT) | 0 or 1 |  | |
| ***Liver and GIT interaction*** | | | |
| BILE_SWITCH (activ. switch bile excretion of CD) | 0 or 1 | 0 or 1 | |
| BILE_SWITCHAM (Act. switch bile excreted CD-OH) | 0 or 1 | 0 or 1 | |
| SWITCH_CONV (activate conversion of CD-OH TO CD in GIT) | 0 or 1 |  | |
| ***Entero hepatic constant (from GIT tract into the liver for parent and metabolism compounds (h-1)*** | | | |
| NONREABSWITHCHRAT (Switch entero rat) | 0 or 1 | - | |
| ENTEROSWITCH (activation switch for CD) | 0 or 1 | 0 or 1 | |
| ENTEROSWITCHMET (activation switch for CD-OH) | 0 or 1 | 0 or 1 | |
| A (fraction of amount in the GIT in the portal circulation) | | | |
| EXTGITSWITCH (active. transmural switch GIT for CD) | 0 or 1 | 0 or 1 | |

**3. Supplementary Figures**

# **PBPK model development and calibration for rat (Figures S1 to S3)**


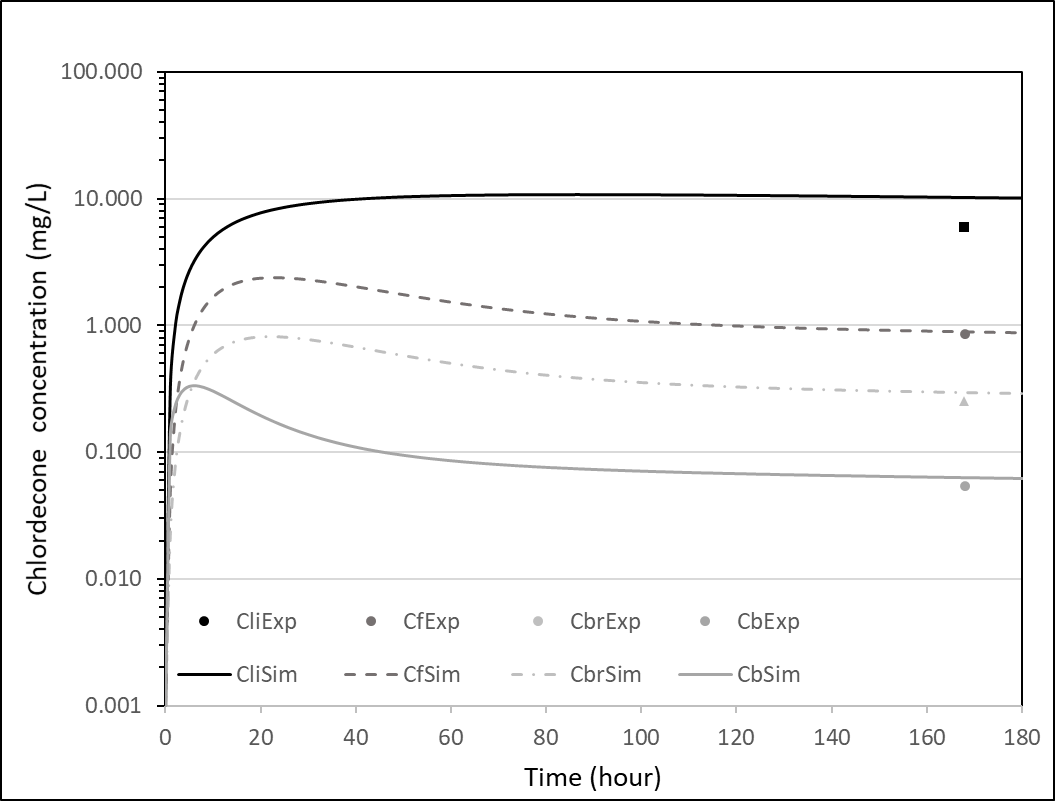


**Figure S1**: Simulation profile following a single exposure to single oral dose of 1 mg of chlordecone/kg bw. The experimental measurement was performed 7 days (168 hours) post exposure (Bungay et al. 1981). The legend corresponded Cli (liver), Cf (adipose tissue), Cbr (brain) and Cb (blood), the Sim correspond to the simulation profile and Exp to the experimental measurement of the chlordecone concentration.


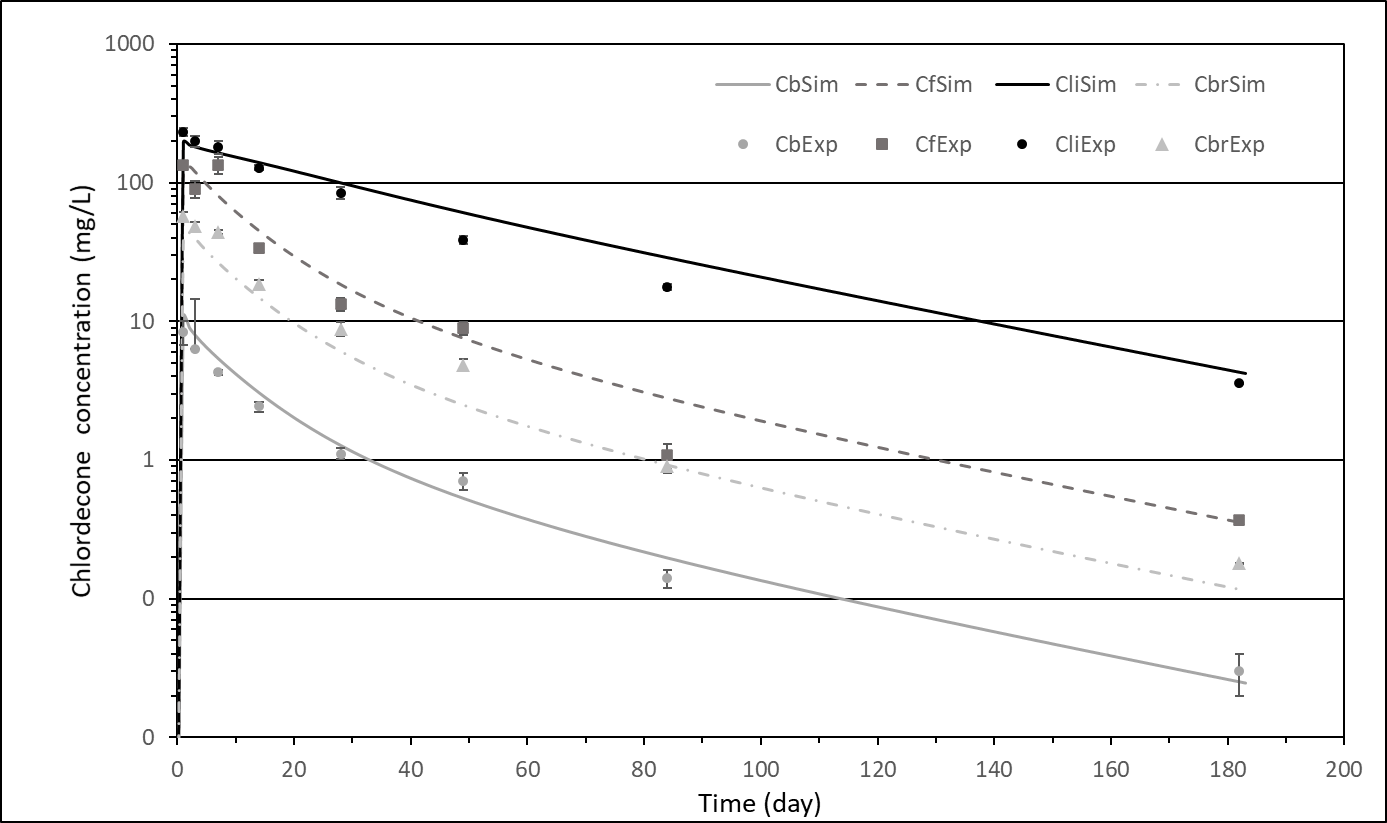


**Figure S2**: Distribution of chlordecone in blood, brain, liver and adipose tissues following a single oral dose of 40 mg CD/kg bw. The legend is concentration in blood (Cb), brain (br), liver (li) or adipose tissue (f), simulated (_Sim) or experimental measured (Egle et al. 1978) _Exp). The y axis is in chlordecone concentration (mg/L) and the x axis represent time as a day.


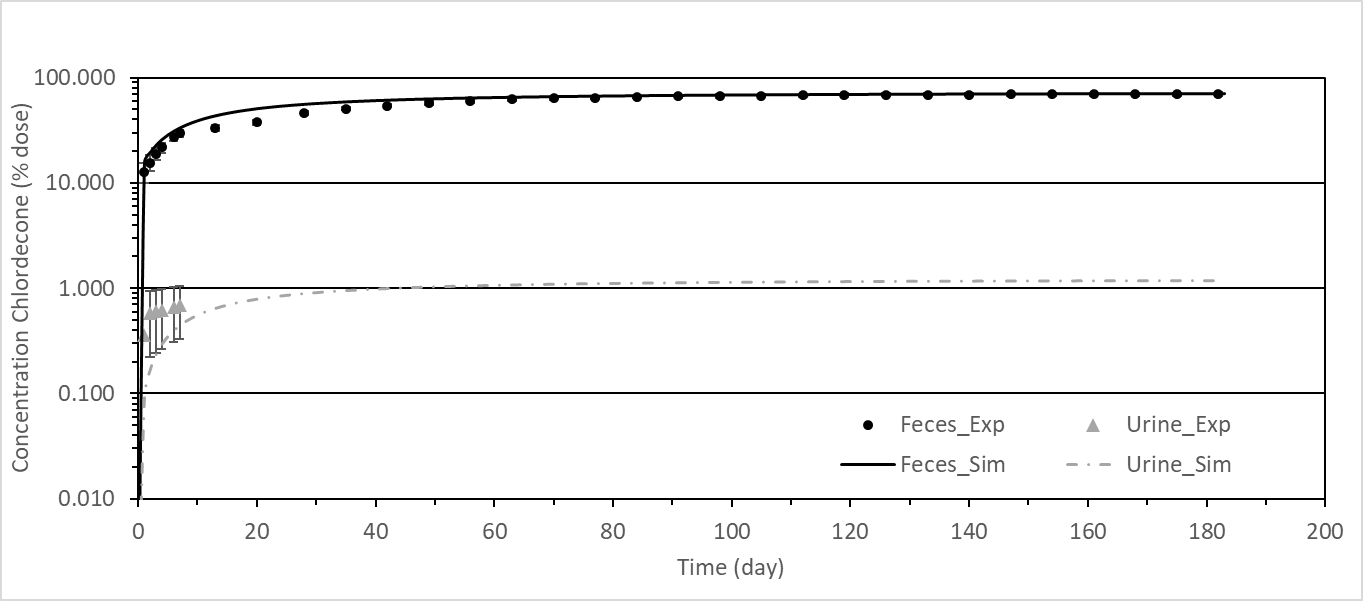


**Figure S3**: Elimination profile of chlordecone in urine and feces following a single oral exposure to 40 mg CD/ kg bw (Egle et al. 1978). The concentration of chlordecone is expressed in percentage dose. The legend corresponds to simulated curve (_Sim) and experimental data points measured (_Exp).

**PBPK model development and calibration for human (Figures S4)**


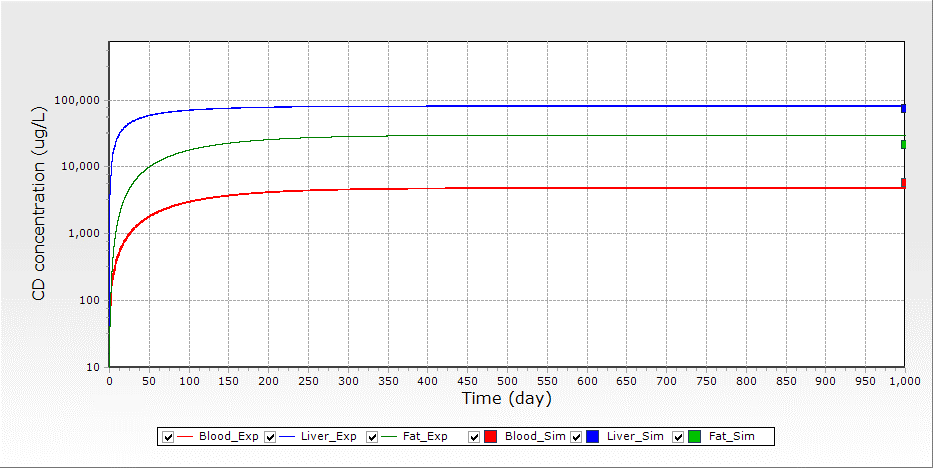


**Figure S4**: Distribution of chlordecone following a subchronic daily exposure until steady state corresponding the condition of chemicals workers chronically exposed. Simulation profiles correspond to a daily oral exposure to 0.19 mg/kg bw/d for 1000 days 7day /week. Lines and dots represent blood in red, liver in blue and adipose tissue in green. _Sim is the simulation with the model and _Exp represent biomonitoring measurement of chlordecone in time (Cohn et al. 1976).

## **Predictability of the rat model (Figures S5 to S17)**


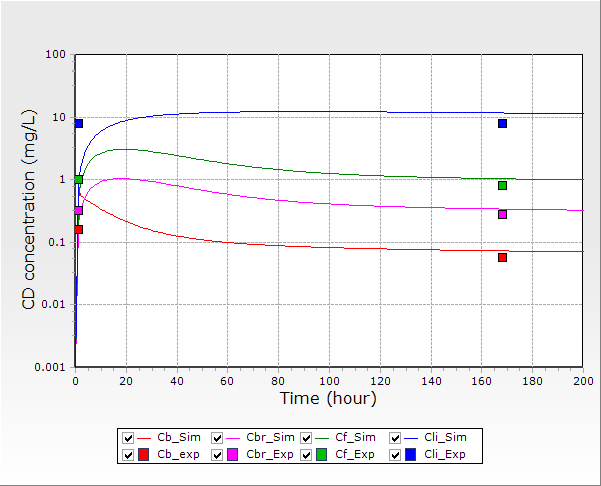


**Figure S5:** distribution of chlordecone following a single intravenous exposure to 1 mg Cd/kg bw. The legend is for blood (Cb), brain (Cbr), adipose tissue (Cf) and liver (Cli). The _Sim is the simulation profile and _Exp the experimental data measured. The x axis is expressed in chlordecone concentration (mg/L) and the x axis is in time hour. The experimental datA came from Bungay et al (1981).


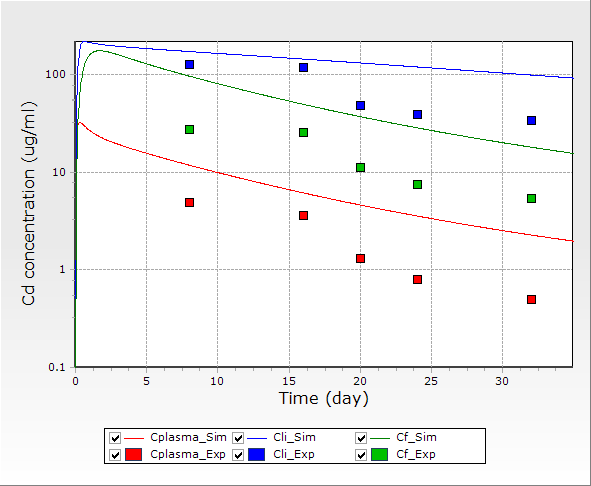


**Figure S6:** distribution of chlordecone following a single oral exposure to 50 mg CD/kg bw. The legend is for plasma concentration (Cplasma), adipose tissue (Cf) and liver (Cli). The _Sim is the simulation profile and _Exp the experimental data measured. The x axis is expressed in chlordecone concentration (mg/L) and the x axis is in time hour. The experimental dat came from Hewitt et al (1981).


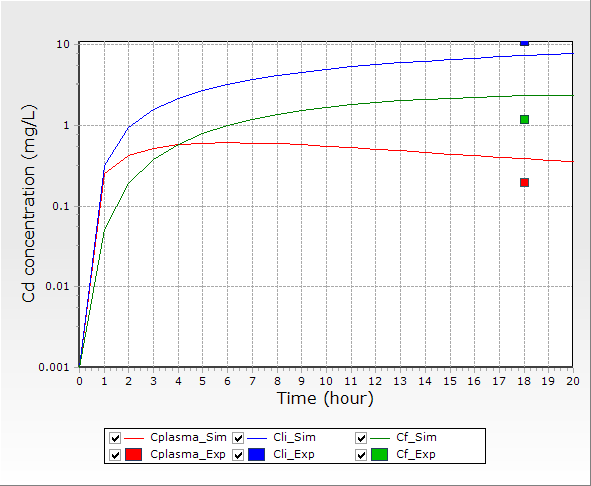


**Figure S7**: distribution of chlordecone following a single oral exposure to 1 mg CD/kg bw. The legend is for blood (Cplasma), adipose tissue (Cf) and liver (Cli). The _Sim is the simulation profile and _Exp the experimental data measured. The x axis is expressed in chlordecone concentration (mg/L) and the x axis is in time hour. The experimental data came from Plaa et al (1987).


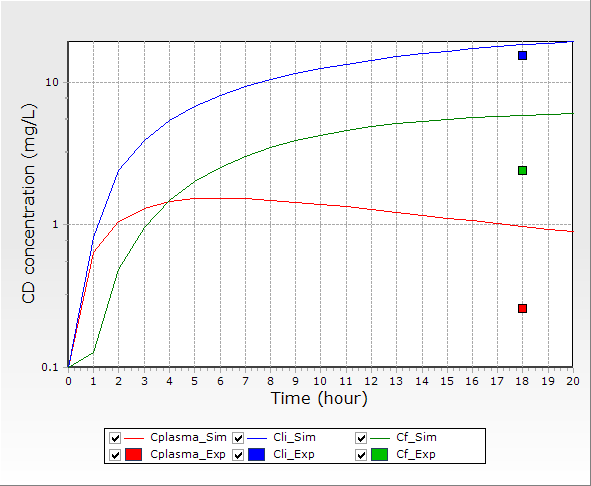


**Figure S8**: distribution of chlordecone following a single oral exposure to 2.5 mg CD/kg bw. The legend is for blood (Cplasma), adipose tissue (Cf) and liver (Cli). The _Sim is the simulation profile and _Exp the experimental data measured. The x axis is expressed in chlordecone concentration (mg/L) and the x axis is in time hour. The experimental data came from Plaa et al (1987).


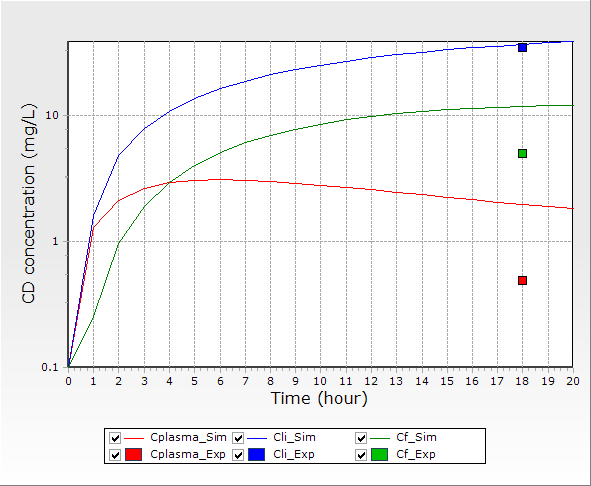


**Figure S9:** Distribution of chlordecone following a single oral exposure to 5 mg CD/kg bw. The legend is for blood (Cplasma), adipose tissue (Cf) and liver (Cli). The _Sim is the simulation profile and _Exp the experimental data measured. The x axis is expressed in chlordecone concentration (mg/L) and the x axis is in time hour. The experimental data came from Plaa et al (1987).


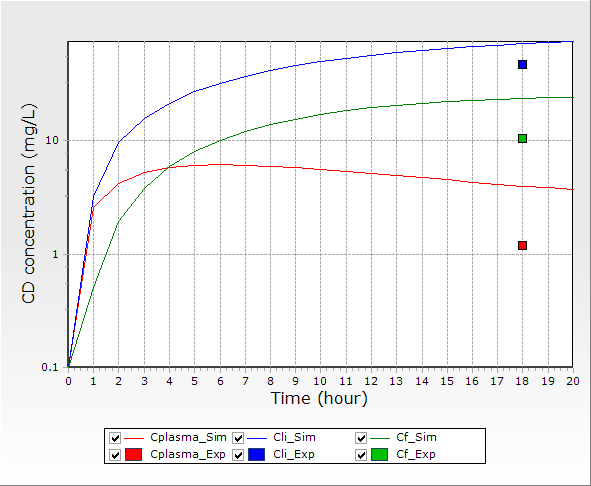


**Figure S10:** Distribution of chlordecone following a single oral exposure to 10 mg CD/kg bw. The legend is for blood (Cplasma), adipose tissue (Cf) and liver (Cli). The _Sim is the simulation profile and _Exp the experimental data measured. The x axis is expressed in chlordecone concentration (mg/L) and the x axis is in time hour. The experimental data came from Plaa et al (1987).


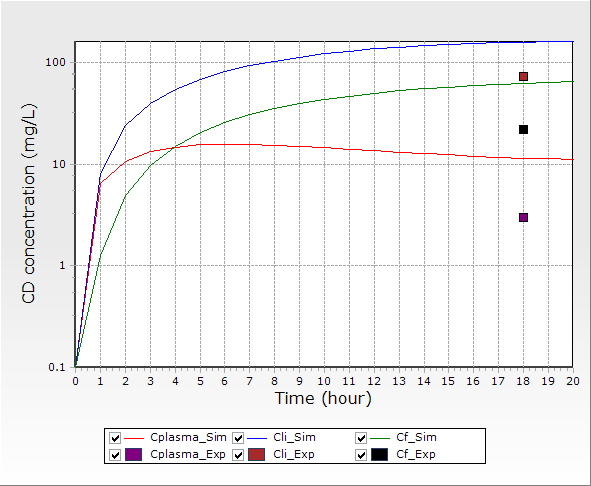


**Figure S11:** Distribution of chlordecone following a single oral exposure to 25 mg CD/kg bw. The legend is for blood (Cplasma), adipose tissue (Cf) and liver (Cli). The _Sim is the simulation profile and _Exp the experimental data measured. The x axis is expressed in chlordecone concentration (mg/L) and the x axis is in time hour. The experimental data came from Plaa et al (1987).


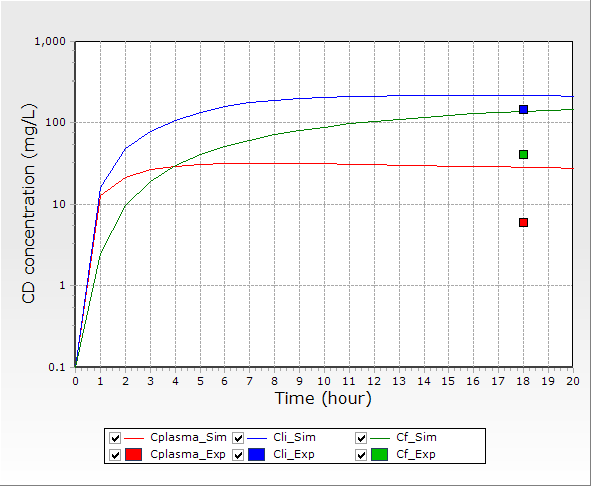


**Figure S12:** Distribution of chlordecone following a single oral exposure to 50 mg CD/kg bw. The legend is for blood (Cplasma), adipose tissue (Cf) and liver (Cli). The _Sim is the simulation profile and _Exp the experimental data measured. The x axis is expressed in chlordecone concentration (mg/L) and the x axis is in time hour. The experimental data came from Plaa et al (1987).


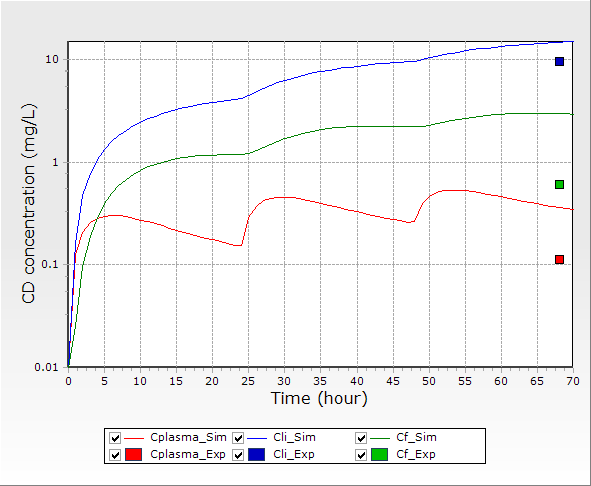


**Figure S13:** Distribution of chlordecone following a single oral exposure to 0.5 mg CD/kg bw for 3 days and sample after 66 hours (18hours post last exposure. The legend is for blood (Cplasma), adipose tissue (Cf) and liver (Cli). The _Sim is the simulation profile and _Exp the experimental data measured. The x axis is expressed in chlordecone concentration (mg/L) and the x axis is in time hour. The experimental data came from Plaa et al (1987).


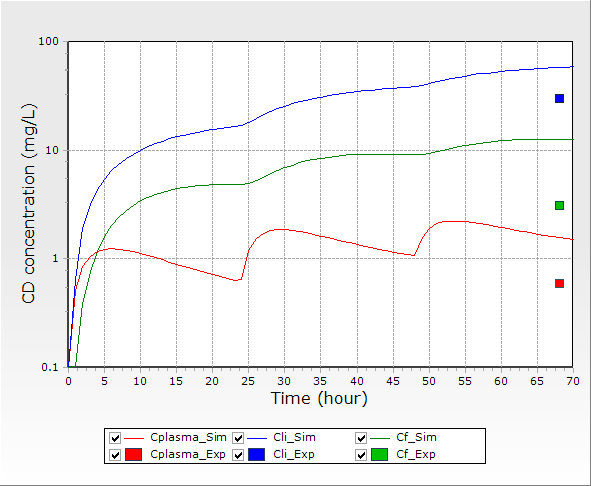


**Figure S14:** Distribution of chlordecone following a single oral exposure to 0.5 mg CD/kg bw for 3 days and sample after 66 hours (18hours post last exposure. The legend is for blood (Cplasma), adipose tissue (Cf) and liver (Cli). The _Sim is the simulation profile and _Exp the experimental data measured. The x axis is expressed in chlordecone concentration (mg/L) and the x axis is in time hour. The experimental data came from Plaa et al (1987).


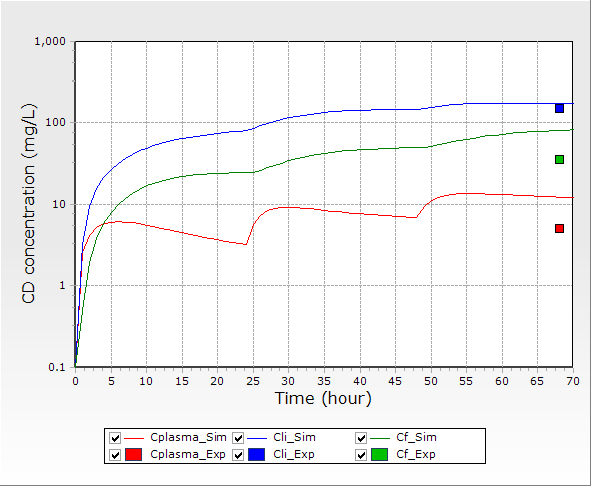


**Figure S15:** Distribution of chlordecone following a single oral exposure to 0.5 mg CD/kg bw for 3 days and sample after 66 hours (18hours post last exposure. The legend is for blood (Cplasma), adipose tissue (Cf) and liver (Cli). The _Sim is the simulation profile and _Exp the experimental data measured. The x axis is expressed in chlordecone concentration (mg/L) and the x axis is in time hour. The experimental data came from Plaa et al (1987).


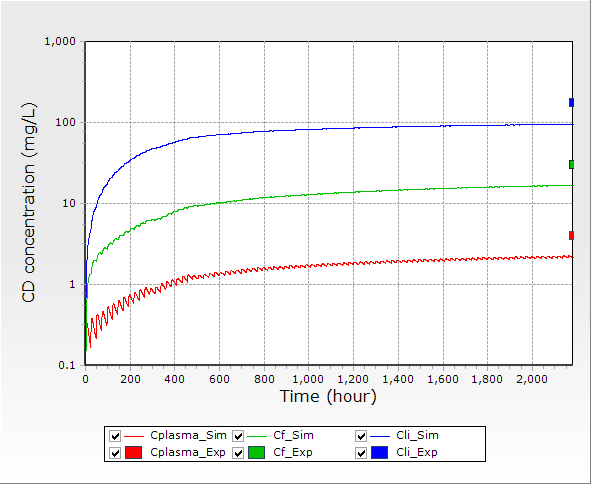


**Figure S16**: Distribution of chlordecone following a single oral exposure to 0.375 mg CD/kg BW (based on 10 ppm in a diet and 10g/100g bw animal eat, 250 g of bw) for 90 days and sample 24 post last exposure at after day 91 (2184 hours of the beginning the treatment). The legend is for blood (Cplasma), adipose tissue (Cf) and liver (Cli). The _Sim is the simulation profile and _Exp the experimental data measured. The x axis is expressed in chlordecone concentration (mg/L) and the x axis is in time hour. The experimental data came from Linder et al., (1983).


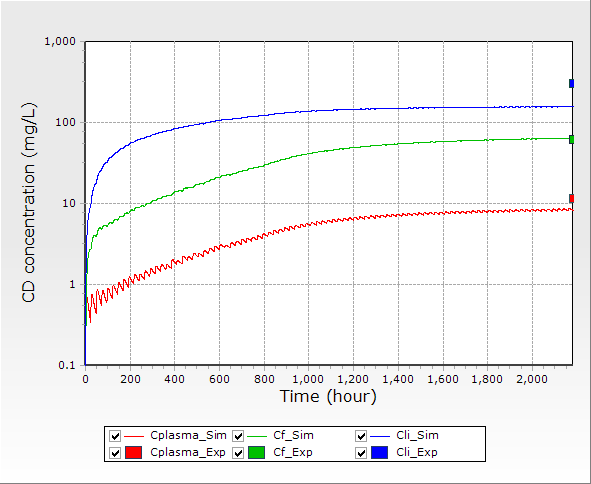


**Figure S17**: Distribution of chlordecone following a single oral exposure to 0.750 mg CD/kg bw (based on 30 ppm in a diet and 10g/100g BW animal eat, 250 g of bw) for 90 days and sample 24 post last exposure on day 91 (2184 hours of the beginning the treatment). The legend is for blood (Cplasma), adipose tissue (Cf) and liver (Cli). The _Sim is the simulation profile and _Exp the experimental data measured. The x axis is expressed in chlordecone concentration (mg/L) and the x axis is in time hour. The experimental data came from Linder et al., (1983).

## **Sensitivity analysis of human model**


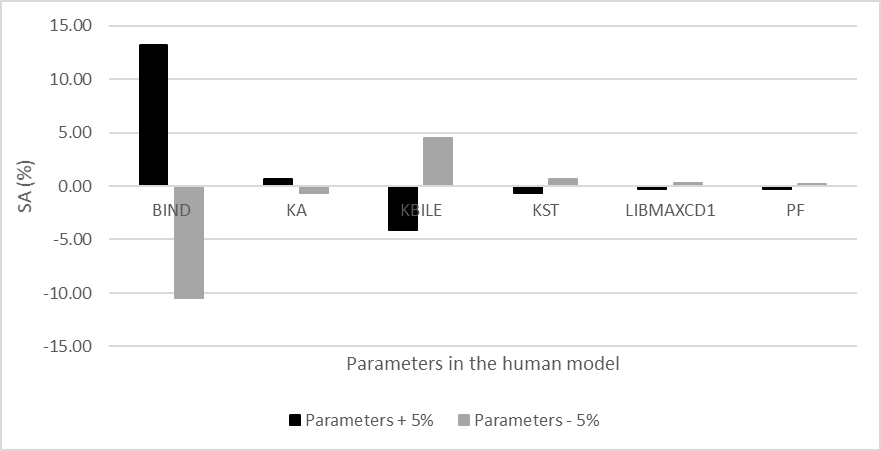


**Figure S18**: Results of the percentage of sensitivity analysis (SA %) of parameters used in the human PBPK model for chlordecone. Only SA% ≥ 0.3 % are presented here in this graph where Bind) fraction of CD bind to lipoprotein in blood, (KA) oral absorption, (KBILE) 1^er^ order function elimination of DC in bile, (KST) transit of unabsorbed CD in the GIT, (LIBMAXCD1) amount of protein binding CD1 in liver, (PF) partition coefficient adipose tissue/blood.

# **4. Parameters and variables symbols used in the models (units)**

Ali = Amount of chemical in a liver cellular matrix sub-compartment (mmole)

Alib = Amount of chemical in the hepatic tissue blood sub-compartment (mmole)

At = Amount of chemical in the tissue cellular matrix sub-compartment (mmole)

Atb = Amount of chemical in the tissue blood sub-compartment (mmole)

$A_{urine}$ = Amount of chemical in urine (mmole)

Avenous = Amount of CD in blood (mmole)

BW = Body weight in (kg)

Ca = Arterial concentration (mmol/l)

Cafree = Concentration of free chemicals in blood (mmol/l)

Cb = Blood systemic venous concentration (mmol/l)

Cli = Liver sub-compartment concentration (mmol/l)

Clib = Liver tissue blood sub-compartment concentration (mmol/l)

Clifree = Free chemical concentration in the liver compartment (mmol/ l)

Cluri = Urinary clearance (ml/day)

Ct = Tissue matrix sub-compartment concentration (mmol/l)

Ctb = Tissue blood sub-compartment concentration (mmol/l)

dAlib/dt= Variation of the amount of chemical in the hepatic blood compartment with time (mmol/day)

dAportal/dt = Variation transfer of chemical from the lumen to the systemic circulation with time (mmol/day)

dAtb/dt = Variation of the amount of chemical in blood sub-compartment with time (mmol/day)

KDLi = affinity constant of chemicals in liver for protein sequestration (mmol/l)

dAuri/dt= Rate of urinary elimination (mmol/day)

Difflipo= Amount of chemicals in the ELPLC (mmole)

dLumen/dt = Rate of CD remaining in the lumen cavity (mmol/day)

free = Fraction of chemicals free in blood circulation (unitless)

Kabs = Oral absorption (day-1)

Kfin = First order constant of chemicals coming in the deep fat compartment (day ^-1^)

Kfout = First order constant of chemicals going out the deep fat compartment (day ^-1^)

Kliout = First order constant of chemicals going out liver into the ELPLC (day ^-1^)

Klipoi = First order constant of chemicals coming in the ELPLC from arterial blood (day ^-1^)

Klipoo = First order constant of chemicals going out the ELPLC into blood circulation (day ^-1^)

Klipooli= First order constant of chemicals going out the ELPLC into the liver (day^-1^)

Kst = First-order rate constant of elimination by the GIT (day-1)

Kmet = First order fraction elimination (day-1)

LiBmaxCD1= Concentration of protein CD1 in the liver compartment (mmol/l)

LiBmaxCD2= Concentration of protein CD2 in the liver compartment (mmol/l)

Lipoi = Amount enter in the ELPLC (mmole)

Lipolo = Amount of chemical leaving liver into ELPLC (mmole)

Lipoo = Amount of chemical leaving the ELPLC into the blood circulation (mmole)

Lipooli = Amount of chemical leaving the ELPLC into the liver (mmole)

Lumen = Amount of CD in the GIT (mmole)

Lymph = amount of chemical in lymph (mmole)

Portal = Amount of chemical in portal vein with time (mmole)

PAli = Liver tissue permeability (PAliF × Qli) (l/day)

PAt = tissue permeability (l/ day)

Pli = Liver/blood partition coefficient (unitless)

Pt = Partition coefficient in tissue compartment (unitless)

Qc = Cardiac output (l/day)

Qcc = Cardiac output (l/day/kg)

Qli = Liver tissue blood flow (Qli0 × Qc) (l/day)

Qt = Blood flow in the tissue compartment (l/h)

Qtf = Blood flw fraction of tissue compartment (unitless)

Vb = Fraction blood volume (liter)

Vli = liver volume (liter)

Vt0 = Fraction volume of the cellular matrice tissue (unitless)

Vt = Volume of the cellular matrix tissue sub-compartment (liter)

Vtb0 = Fraction volume tissue/blood subcompartment (uniteless)

Vtb = Volume of the tissue/blood sub-compartment (liter)

# **5. Equations used for human model and their units**

#### **Initial condition for the simulation**

Y0 = 10 Year at beginning of simulation

GYR = Y0 + T/365 TIME IN TERM OF YEAR

#### **Male equation of bw in kg**

ABWM=0.00058

BBWM=0.0948

CBWM=4.8434

DBWM=2.278

BWM_kg = ((ABWM*GYR**3)-(BBWM*GYR**2)+(CBWM*GYR) +DBWF)

#### **Female equation of bw in kg**

ABWF=0.0006

BBWF=0.0912

CBWF=4.3200

DBWF=3.6520

BWF_kg = ((ABWF*GYR**3)-(BBWF*GYR**2)+(CBWF*GYR)+DBWF)

BWT= (BWF_kg*FEMALE+BWM_kg*MALE) !BODY WEIGHT IN KG

## ***Cardiac output (l/day)***

${QC}^{*}= QCC*60*{(BW)}^{0.75}$

## ** Fraction of volume and blood flow were calculated using polynomial equation of Luecke et al., 2007*

## ***Volume compartment (l)***

$$Vt=Vt0\times Vt$$

## ***Tissue blood Volume compartment (l)***

$$Vtb=Vtb0\times Vt$$

## ***Blood flow in compartment (l/day)***

$$Qt=Qtf\times Qc$$

## ***Exchange in the Extracellular lipoprotein / lymphatic circulation (ELPLC) (mmol/day)***

### Rate change of CD enter in the ELPLC from blood compartment

$$RLipoi=\frac{d Lipoi}{dt}=Klipoi\times Avenousfree$$

### Rate change of CD leaving the ELPLC to blood compartment

$$RLipoo=\frac{d Lipoo}{dt}=Klipoo\times Difflipo$$

### Rate change of CD leaving the ELPLC compartment into liver tissue

$$RLipooli=\frac{dLipooli}{dt}=Kliout\times Difflipo$$

### Rate change of CD leaving liver tissue in the ELPLC compartment

$$RLipolo=\frac{dLipoli}{dt}=Kliout\times ali$$

### Rate change of CD in the ELPLC compartment (mmole)

$$Difflipo=Lipoi-Lipoo-Lipooli+Lymph+lymph1$$

# ***Blood compartment (mmol/day or mmol/l)***

$RAvenous=\frac{dtAvenous}{dt} =(\left( Qlu\times Vclu \right)+\left( Qbr\times Cbrb \right)+\left( Qf\times Cfb \right)+\left( Qre\times Creb \right)+(EXPLV+RAURI+RLIPOO-RLIPOI-RAGITW))-Qc\times Cb\times Free$

$$Cb=Avenous/Vb$$

$$Ca=Cb$$

$$Cafree=Cb\times free$$

# ***Tissue compartment for diffusion limited (brain, adipose tissue, and rest of the body)***

Tissue blood sub-compartment:

$\frac{dAtb}{dt}=Qt\left( Cafree-Ctb \right)-PAt\left( Ctb-\frac{Ct}{Pt} \right)$

$$Ctb=\frac{Atb}{Vtb}$$

Tissue cellular matrices:

$$\frac{dAt}{dt}=PAt\left(Ctb-\frac{Ct}{Pt}\right)$$

$$Ct=\frac{At}{Vt}$$

$$Ct=\frac{Ct}{Pt}$$

# ***Liver tissue compartment (liver) (mmol/day or mmol/l)***

Tissue blood sub-compartment:

$$\frac{dAlib}{dt}=Qli\left( Cafree-Clib \right)-PAli\left( Clib-\frac{Clifree}{Pli} \right)+RPORTAL+RPORTAL1$$

$$Clifree=Cli-\left( Clifree\times Pli \right)+\left[ \frac{LiBmaxCD1\times Clifree}{Kdli1+Clifree} \right]+\left[ \frac{LiBmaxCD2\times Clifree}{Kdli2+Clifree} \right]$$

$Clib(mmol/l)=\frac{Alib}{Vlib}$

Tissue cellular matrices:

$\frac{dAli}{dt}=PAli\left( Clib-\frac{Cli}{Pli} \right)-\frac{dAmet}{dt}-\frac{dLipooli}{dt}-\frac{dLipolo}{dt}$

$$Cli=\frac{Ali}{Vli}$$

# ***Gastrointestinal absorption and distribution of CD to the portal and lymphatic circulation***

Amount of CD remaining in the GIT lumen cavity (mmol/day):

$\frac{dLumen}{dt}\left( \frac{nmol}{hour} \right)=-\left[ \left( Kst+Kabs \right)\times Lumen \right]+RTRANSIT+RBILEOUT+RCONVERAMCD$

*Lumen* = Amount of CHLORDECONE remaining in the GI tract

*Intake* = Rate of intake of CHLORDECONE during a repetitive exposure

A = fraction of portal absorption

B= Fraction of lymphatic absorption (B = 1-A)

Amount of CD eliminated in the feces (mmol/day):

$\frac{dFeces}{dt}=Kst*Lumen$

Absorption rate of CD by the liver via portal circulation (mmol/day):

$\frac{dPortal}{dt}=Kabs\times A\times Lumen$

Absorption rate of CD in the lymphatic circulation (mmol/day):

$\frac{dLymph}{dt}=Kabs\times B\times Lumen$

## ***Urinary elimination (l/day)***

$$Rauri=\frac{d urine}{dt}=Cluri\times Cbfree$$

## ***Enterohepatic circulation and metabolism***

#### Rate of chlordecone eliminated in bile (mmol/day)

| KBILE | = | Elimination rate of CD in bile (day^-1^) |
| --- | --- | --- |
| BILE_SWITCH | = | BILE_SWITCH=0 (rat) no excretion; BILE_SWITCH=1 bile excretion |

$$Rabile=(Kbile\times Cfllir\times Vli)\times Bile\_switch$$

#### Amount metabolized (mmol/day)

| KELMET | = | Metabolic clearance formation of CD-OH (L/day) |
| --- | --- | --- |
| Ram | = | Rate change of metabolite in liver (mmol/day) |

$$Ram=Kelmet\times Cfllir$$

#### Metabolism elimination in bile (mmol/day)

| KBILEAM | = | Biliary elimination constant of the metabolism (1/day) |
| --- | --- | --- |
| BILE_SWITCHAM | = | Switch transfer of biliary elimination (0)= no, (1) yes |
| Am | = | Amount of metabolite produce (mmole) |

$$Rambileout=Kbileam\times Am\times Bile\_swithcham$$

#### Conversion rate of CD-OH or CD-O-G into CD in the GIT (mmol/day)

| KCONVAMCD | = | Conversion constant rate of CD-OH or CD-O-G into CD (1/day) |
| --- | --- | --- |
| SWITTCH_CONV | = | Switch activated the conversion (0) no, (1) yes |

$$Rconveramcd=Ambileout\times Kconvamcd\times Swittch\_conv$$

## **6. References**

Belfiore CJ, Yang RSH, Chubb LS, Lohitnavy M, Lohitnavy OS, Andersen ME (2007) Hepatic sequestration of chlordecone and hexafluoroacetone evaluated by pharmacokinetic modeling. Toxicology 234(1-2):59-72 doi:10.1016/j.tox.2007.02.002

Bungay PM, Dedrick RL, Matthews HB (1979) Pharmacokinetics of halogenated hydrocarbons. AnnNYAcadSci 320:257-270

Bungay PM, Dedrick RL, Matthews HB (1981) Enteric transport of chlordecone (Kepone) in the rat. JPharmacokinetBiopharm 9(3):309-341

Cohn WJ, Blanke RV, Griffith FD, Guzelian PS (1976) Distribution and excretion of Kepone (KP) in humans. vol 71, p 901

Egle JL, Fernandez JB, Guzelian PS, Borzelleca JF (1978) Distribution and excretion of chlordecone (Kepone) in the rat. Drug Metab Dispos 6(1):91-95

Emond C, Birnbaum LS, DeVito M (2004) Physiologically based pharmacokinetic model for developmental exposures to TCDD in the rat. Toxicol Sci 80(1):115-133

GastroPlus (2018) GastroPlus Simulation software for drug discovery and development (version 9.6). Simulation Plus p748

Heatherington AC, Fisher HL, Sumler MR, Waller CL, Shah PV, Hall LL (1998) Percutaneous Absorption and Disposition of [14C]Chlordecone in Young and Adult Female Rats. Environmental Research 79(2):138-155 doi:10.1006/enrs.1998.3862

Krishnan K, Andersen M (2008) Physiologically based pharmacokinetic and toxicokinetic models. In: Hayes AW (ed) Principles and methods of toxicology. vol 5th. CRC Press, New York, p 231-291

Luecke RH, Pearce BA, Wosilait WD, Slikker W, Young JF (2007) Postnatal Growth Considerations for PBPK Modeling. Journal of Toxicology and Environmental Health, Part A 70(12):1027-1037

Shah PV, Fisher HL, Sumler MR, Hall LL (1989) Dermal absorption and pharmacokinetics of pesticides in rats ACS Sympsoum Series 382. Biological Monitoring for pesticide exposure. Measurement, estimation, and risk reduction. American Chemical Society, Washington, DC 1989, p 169-187
